# Maladaptive coping strategies mediate the relationship between perceived differential treatment and sleep health among women

**DOI:** 10.1007/s10865-026-00710-1

**Published:** 2026-09-07

**Authors:** Rupsha Singh, Symielle A. Gaston, Dale P. Sandler, Chandra L. Jackson

**Affiliations:** 1 Epidemiology Branch, National Institute of Environmental Health Sciences, National Institutes of Health, Department of Health and Human Services, 111 TW Alexander Drive, Research Triangle Park, NC 27709, USA; 2 Intramural Program,, National Institute on Minority Health and Health Disparities, National Institutes of Health, Department of Health and Human Services, Bethesda, MD, USA

**Keywords:** Perceived differential treatment, Coping strategies, Sleep, Women

## Abstract

To investigate whether associations between perceived differential treatment based on race-ethnicity and sleep are mediated by coping strategies. We used data from the nationwide Sister Study. In 2008–2012, participants self-reported perceived lifetime differential treatment and completed adapted Coping Orientation to Problems Experienced Inventory (COPE)/Brief COPE questionnaires which assessed healthy/adaptive (e.g., using emotional support) and maladaptive (e.g., self-blame) coping. Sleep duration, poor sleep quality, and insomnia symptoms were self-reported in 2017–2019. Adjusting for sociodemographic and health characteristics, mediation analysis was performed using the Hayes PROCESS macro. Among 31,443 eligible women (mean ± SD age 55.5 ± 8.6 years), the prevalence of everyday (79% among non-Hispanic [NH]-Black, 30% Latina, and 4% NH-White participants) and major (57% NH-Black, 14% Latina, and 2% NH-White) perceived differential treatment varied by race-ethnicity. Among NH-Black women, only maladaptive coping mediated associations between everyday differential treatment and poor sleep quality (indirect effect *b* = 0.01, 95% confidence interval [CI] = 0.00, 0.03) and insomnia symptoms (indirect effect *b* = 0.04, 95% CI = 0.01, 0.07). Neither adaptive nor maladaptive coping mediated (1) associations with major differential treatment among NH-Black women nor (2) associations with differential treatment among Latina women. Among NH-White women, maladaptive coping mediated associations between both everyday and major differential treatment and both poor sleep quality and insomnia symptoms. Associations between everyday differential treatment based on race-ethnicity and insomnia symptoms along with poor sleep quality were stronger among women who reported maladaptive, but not healthy/adaptive, coping strategies. Interventions that address maladaptive coping may mitigate the impact of perceived differential treatment on sleep disturbances.

## Introduction

Poor sleep is a major yet underappreciated contributor to poor mental and cardiovascular health (Lim et al., 2023; Watson et al., 2015). It is estimated that approximately 83.6 million adults in the U.S. regularly get less than the recommended 7 or more hours of sleep per night (Liu, 2016; Panel, 2015). Additionally, differences in sleep health have been observed across racial-ethnic groups, with certain populations experiencing a higher prevalence of sleep-related challenges that may contribute to physical and mental health outcomes (Chen et al., 2015; Johnson et al., 2019). For instance, compared with their non-Hispanic (NH) White counterparts, NH-Black/African American adults habitually have shorter sleep durations, lower sleep efficiency, longer sleep onset latency, and less time in slow wave sleep (i.e., deep sleep), which is considered the most physiologically restorative sleep stage (Grandner et al., 2016; Johnson et al., 2019; Ruiter et al., 2011). Similarly, Latinx adults have higher prevalence of shorter as well as longer (> 9 h) sleep duration, poorer sleep quality, restless sleep, and daytime sleepiness compared with NH-White adults (Roncoroni et al., 2022). These differences in sleep health are associated with a higher risk of chronic conditions including obesity and cardiovascular disease, which are also more prevalent among Latinx and Black/African American adults (Metlock et al., 2026; Tubbs et al., 2022).

There is growing acknowledgment among researchers that observed disparities in sleep health likely stem from a combination of broader societal influences, individual-level factors, and lived experiences (Johnson et al., 2022). Although data on historical and societal influences are minimal, a growing body of evidence underscores the link between experiences of differential treatment based on race-ethnicity and adverse sleep health, including short sleep duration, insomnia symptoms, and increased sleep disturbance (Gaston et al., 2020; Gordon et al., 2020; Hart et al., 2021; Hicken et al., 2013; Mead et al., 2023; Slopen et al., 2016). It is hypothesized that differential treatment may impact sleep by inducing feelings of threat, arousal, and vigilance (Harrell et al., 2003; Hicken et al., 2013); by increasing psychological distress, such as depression, anxiety, anger, and hostility (Beatty et al., 2011; Brondolo et al., 2011; Huynh et al., 2012); or by triggering overactivation of physiological stress response systems (Buckley & Schatzberg, 2005). These hypothesized pathways may lead to maladaptive coping behaviors (e.g., rumination, substance use) that can negatively impact sleep.

Coping, which involves cognitive and behavioral efforts to manage stressors, plays a crucial role in influencing the impact of stress on health outcomes (Carr & Umberson, 2013; Lazarus, 1984). Individuals employ a variety of coping strategies, ranging from adaptive approaches that foster resilience to maladaptive ones that may exacerbate the impact of stressors. The environmental affordances (EA) model provides a theoretical framework to understand how individuals navigate and adapt to their environments, including social contexts marked by differential treatment (Mezuk et al., 2013). This model posits that the environment acts as a source of constraints or stressors (e.g., greater access to tobacco, alcohol, and illicit drugs, limited access to healthy foods) as well as a source of affordances or opportunities (e.g., strong family and community ties, cultural practices that promote resilience) (Mezuk et al., 2013). These environmental contexts interact with coping behaviors influenced by cultural and social norms, as well as individual characteristics like age, sex, and race-ethnicity. For instance, the EA model suggests that exacerbated engagement in unhealthy behaviors (i.e., substance use, eating calorie-dense foods) to cope with exposure to stressors among Black Americans is influenced by residential separation (Schwartz et al., 2022). Such separated neighborhoods often have limited access to healthy foods and recreational activities but more access to tobacco, alcohol, and illicit drugs than other neighborhoods (Vaeth et al., 2017; Williams & Collins, 2001). Exposure to these insalubrious factors may contribute to maladaptive coping behaviors, which in turn exacerbate health risks. This may simultaneously explain—in part, the observed high risk of chronic conditions such as type 2 diabetes and cardiovascular disease among Black Americans (Baez et al., 2023; Gebreab et al., 2017; Xu et al., 2022).

While maladaptive coping behaviors contribute to increased chronic disease prevalence and risk, they may also mask the true burden of mental health conditions. For instance, substance use as a coping mechanism may temporarily alleviate distress, leading to an equal or lower reported prevalence of mental health conditions such as depression and anxiety among Black Americans compared with White Americans, a phenomenon known as the “Black-White depression paradox” (Keyes, 2009). However, this paradox does not necessarily indicate better mental health; rather, it suggests that distress may manifest in other ways, including physiological disruptions such as poor sleep. Sleep disturbances, including short sleep duration and insomnia symptoms, have been linked to both chronic disease risk and psychological distress, yet they may be underrecognized in populations where mental health conditions are less frequently reported (Goldstein et al., 2020; Williams et al., 2007). This underscores the importance of examining how coping responses, shaped by environmental contexts, influence sleep health. Overall, the EA model (Mezuk et al., 2013) suggests that lived experiences in surrounding environments marked by perceived differential treatment, may limit healthy/adaptive coping resources or promote maladaptive coping, thereby intensifying the adverse health impacts of differential treatment, including on sleep health. Conversely, environments that facilitate healthy/adaptive coping resources and support may mitigate the adverse effects of differential treatment on sleep health.

Very few studies on perceived differential treatment, coping, and sleep have focused on women and examined associations within racial-ethnic groups. However, such investigations are warranted given well-documented sex differences in sleep health. Women report poorer subjective sleep quality and a higher prevalence of insomnia symptoms compared to men, despite often having longer total sleep time and greater slow-wave activity (Mallampalli & Carter, 2014; Mong & Cusmano, 2016). These sex differences are influenced by both biological factors, such as hormonal fluctuations across the menstrual cycle, pregnancy, and menopause, and psychosocial factors, including greater stress reactivity and caregiving demands (Mallampalli & Carter, 2014; Mong & Cusmano, 2016). Moreover, women face persistent barriers that affect their access to resources, opportunities, and healthcare, contributing to stress and health challenges (P. Homan 2024a, b). NH-Black/African American and Hispanic/Latina women may be particularly susceptible to poor coping than non-Hispanic White men or women due to a combination of differential treatment based on race and sex (Everett et al., 2010; Spates et al., 2020; Thomas et al., 2008). This dual burden may uniquely shape women’s stress experiences and coping responses (Everett et al., 2010; Spates et al., 2020; Thomas et al., 2008), influencing sleep in ways that may differ from their male counterparts, thus underscoring the need for comprehensively investigating drivers of sleep health, including perceived differential treatment and coping, by sex (Gaston et al., 2020; Jackson et al., 2020).

Research has linked both perceived differential treatment and poor sleep with maladaptive coping, suggesting a bidirectional relationship. Among adolescents from various backgrounds in the United States, better sleep was associated with greater use of active coping strategies, which in turn contributed to improved well-being (Wang & Yip, 2020). Similarly, a study in Japan found that workers with insomnia symptoms were more likely to engage in maladaptive coping strategies compared with those without insomnia (Otsuka et al., 2022). Among Latinx adults, coping strategies influenced the relationship between differential treatment and sleep, with passive-active coping (e.g., accept unfair treatment as a fact of life) linked to poorer sleep quality under everyday differential treatment, and active coping (e.g., try to do something about unfair treatment) associated with greater sleep disturbances under major lifetime differential treatment (Majeno et al., 2022). While prior research suggests that poor sleep may also impair coping capacity and exacerbate perceptions of differential treatment, we use the EA model as a guiding theoretical framework to conceptualize differential treatment as an external stressor that elicits coping responses, which in turn shape health outcomes such as sleep. Prior analyses of the Sister Study demonstrated that perceived differential treatment based on race-ethnicity was directly associated with multiple sleep dimensions (Gaston et al., 2020). However, the pathways explaining these associations remain unclear. Our study builds on this work by testing coping strategies as mediators of the differential treatment–sleep relationship. By incorporating coping within the EA model framework, we move beyond documenting associations to evaluating the mechanisms through which differential treatment may affect sleep health. Moreover, despite prior research examining coping and sleep in different contexts, no studies to date have examined the potential mediating role of adaptive versus maladaptive coping strategies in associations between everyday and major differential treatment based on race-ethnicity in relation to sleep health among a cohort of NH-White, NH-Black, and Hispanic/Latina U.S. women. Therefore, we sought to investigate these mediation pathways to better understand how coping mechanisms may influence the association between perceived differential treatment and sleep health (see Fig. 1 for conceptual model). Although the sample is predominantly NH-White, inclusion of NH-White participants allows for a more comprehensive characterization of the prevalence and associations of differential treatment across three racial-ethnic groups and enables examination of whether coping pathways linking differential treatment to sleep operate similarly or differently within each racial-ethnic group. We hypothesized that perceived differential treatment is associated with higher maladaptive coping strategies and lower adaptive coping strategies. In turn, maladaptive coping strategies are associated with poorer sleep, while adaptive coping strategies are associated with more favorable sleep.

## Methods

### Data source: the Sister Study

The Sister Study is a prospective cohort study of 50,884 women in the United States, including Puerto Rico, ages 35 to 74 years at enrollment between 2003 and 2009. Eligible participants, who were free of breast cancer at baseline but had a sister previously diagnosed with the disease completed self-administered questionnaires, underwent a home visit for the collection of biologic specimens, and participated in a computer-assisted telephone interview conducted in either English or Spanish. Detailed follow-up questionnaires were administered every 2 to 3 years post-enrollment, and less detailed follow-up questionnaires were administered annually. Written informed consent was obtained from all participants, and the Institutional Review Board of the National Institute of Environmental Health Sciences oversees the Sister Study. We used data release 10.1, which included follow-up information until 2019.

### Study population

To be eligible for the current study, participants needed to self-identify as NH-White alone (referred to as NH-White), NH-Black alone (referred to as NH-Black), or Hispanic/Latina (of any race), to ensure sufficient sample size for stable within-group analyses. Race-ethnicity was classified in accordance with Office of Management and Budget (OMB) guidelines, under which NH-White includes individuals of Middle Eastern descent. Additional eligibility criteria included having complete data on differential treatment based on race-ethnicity (collected at the first detailed follow-up from 2008 to 2012), coping strategies (also collected during the first-detailed follow-up), and at least one sleep dimension (i.e., sleep duration, poor sleep quality, or insomnia symptoms), collected during the fourth-detailed follow-up (2017–2019). Exclusions were applied simultaneously; because some participants met multiple criteria, exclusion counts are not mutually exclusive, resulting in a final analysis sample of 31,443 participants (see Fig. 2). The final sample included 28,619 NH-White women, 1838 NH-Black women, and 986 Hispanic/Latina women.

### Exposure: perceived differential treatment based on race-ethnicity

During the first detailed follow-up (2008–2012), participants self-reported whether they had ever experienced unfair treatment attributed to their race or ethnicity. Everyday differential treatment included three items (Williams et al., 1997): treated unfairly in receiving service at a store or restaurant; treated as less intelligent, worthy, or honest than others; and experienced people acting as if they are afraid of you. Major differential treatment was assessed using the following three items: treated unfairly in home renting, buying, or mortgage lending; treated unfairly in being stopped, searched, or threatened by police; and treated unfairly in job hiring, promotion, or firing. Each form of differential treatment based on race-ethnicity was defined as a report of any of the three items, consistent with prior studies on perceived differential treatment and sleep (Gaston et al., 2020).

### Outcome: self-reported sleep duration, poor sleep quality, and insomnia symptoms

At the fourth follow-up (2017–2019), participants self-reported their typical bedtime and wake-up time for both weekdays/workdays and weekends/non-workdays, from which average weekly sleep duration was calculated. Based on the National Sleep Foundation categories, average sleep duration per day was defined as either short (< 7 h), recommended (7–9 h), or long (> 9 h) (Hirshkowitz et al., 2015). Sleep disturbances (henceforth, referred to as poor sleep quality) were assessed using the eight-item PROMIS Sleep Disturbance questionnaire (Buysse et al., 2010), and categorized as “none to slight,” “mild,” and “moderate to severe.” Insomnia symptoms were defined by reports of either difficulty initiating sleep (taking > 30 min to fall asleep on average) or difficulty maintaining sleep (waking up in the middle of the night or early morning) three or more times per week, compared to reporting neither symptom.

### Potential mediators: adaptive and maladaptive coping strategies

Self-reported coping strategies were assessed at the first detailed follow-up (2008–2012) using nine items adapted from the Coping Orientation to Problems Experienced Inventory (COPE) and Brief-COPE scales (Carver, 1997; Carver et al., 1989). The items were categorized as adaptive or maladaptive coping strategies based on factors proposed by Paukert and colleagues (Paukert et al., 2009). Adaptive coping included five items: use of emotional support (get emotional support/comfort/understanding from others), active coping (take action to try make situation better), acceptance (learn to live with things), humor (try to laugh or make fun of the situation), and growth (try to grow as a person from the experience). Maladaptive coping included four items: behavioral disengagement (give up trying to deal with things or trying to cope), denial (refuse to believe that things have happened), self-blame (criticize or blame self), and venting (express negative feelings). The responses were measured on a Likert-type scale, ranging from 1 (“not at all”) to 4 (“a lot”). Items were summed to create scores that ranged from 5 to 20 for adaptive coping and 4–16 for maladaptive coping.

### Potential confounders

Potential confounding variables included sociodemographic, health behavior, and clinical characteristics that were selected a priori based on prior literature (Park et al., 2010; Whinnery et al., 2014). Sociodemographic variables were measured at baseline and included self-reported age, educational attainment, employment, annual household income, and marital status. Health behavior variables included self-reported sleep medication use, assessed at the fourth follow-up. Clinical characteristics included body mass index (assessed at baseline with measured height and weight), self-reported depressive symptoms (assessed at first follow-up), and self-reported general health status (assessed at fourth follow-up).

### Statistical analyses

#### Primary analysis.

Parallel mediation analyses were performed using PROCESS macro Model 4 (simple mediation) by Andrew Hayes (Hayes, 2013) in Statistical Package for the Social Sciences (SPSS) Version 29. We used parallel mediation to examine both coping variables simultaneously, thereby accounting for their shared associations while examining how each individually influences sleep health 5–11 years later (Hayes, 2013). The PROCESS macro was used to provide bias-corrected bootstrapped standard errors for the indirect effects and confidence intervals (CIs) for the parallel mediators. Separate parallel mediation models were computed for each form of differential treatment and sleep outcome within each racial-ethnic group. Total, direct, and indirect effects were estimated for sleep duration and poor sleep quality. Total effects for insomnia symptoms could not be estimated because the outcome was binary, and in logistic regression, total effect is not equal to the sum of direct and indirect effects due to the non-linear nature of the model. As a result, the PROCESS macro does not compute or report total effects for binary outcomes to avoid misinterpretation. All models were adjusted for age, marital status, annual household income, educational attainment, employment status, and body mass index at baseline; depressive symptoms at the first detailed follow-up; and general health status and sleep medication use at the fourth detailed follow-up, to preserve temporality and avoid overadjustment (VanderWeele, 2019). Notably, we tested for indirect effects even when total or direct effects were not significant, following Hayes’ recommendation that mediation can still reveal meaningful pathways even in the absence of direct effects (Hayes, 2013). This is particularly relevant for intervention research, as relying on a causal steps framework may overlook mediators that are valuable targets for intervention efforts (O’Rourke & MacKinnon, 2018).

#### Secondary analysis.

Using aforementioned analytic approaches and PROCESS macro Model 7 (moderated mediation), we also performed secondary analyses to test whether educational attainment and household income moderated the mediating effects of coping strategies on the relationship between differential treatment and sleep health 5–11 years later. These socioeconomic factors may shape both access to coping resources and the effectiveness of coping strategies in mitigating differential treatment-related stress, ultimately influencing sleep health (Meyer et al., 2008; Sheehan et al., 2020). Separate parallel moderated mediation was conducted for household income and educational attainment to estimate the index of moderated mediation (IMM), which quantifies whether and how much the strength of the indirect effect varies across levels of a moderator. A significant IMM indicates that the mediating role of coping strategies differs based on socioeconomic status, helping to identify for whom and under what conditions coping strategies are more or less effective in linking differential treatment to sleep health.

## Results

### Participant characteristics

Among the 31,443 included participants, 91.0% self-identified as NH-White, 5.8% as NH-Black, and 3.1% as Hispanic/Latina (Table 1). The mean age was 55.5 years ± 8.6 years, and most (84.0%) were between ages 35 and 64 years. At the fourth follow-up, prevalence of habitual short sleep duration was highest among NH-Black participants (29.8%) followed by Hispanic/Latina (16.2%) and NH-White (10.0%) participants (*p* < 0.001). A similar pattern of moderate-to-severe poor sleep quality was present (12.2% among NH-Black, 11.3% among Hispanic/Latina, and 9.5% among NH-White women, *p* = 0.001). The prevalence of insomnia symptoms was highest among NH-White participants (43.8%) followed by NH-Black (39.9%) and Hispanic/Latina (38.3%) participants (*p* < 0.001). Among NH-Black participants, 78.6% (vs. 3.8% NH-White and 29.7% Hispanic/Latina, *p* < 0.001) reported everyday differential treatment, and 57.1% (vs. 2.2% NH-White and 14.3% Hispanic/ Latina, *p* < 0.001) reported major differential treatment. There were statistically significant racial-ethnic differences in average adaptive coping strategy scores (mean ± SD: 16.2 ± 2.4 for NH-White, 16.2 ± 2.3 for NH-Black, and 16.0 ± 2.6 for Hispanic/Latina participants, *p* = 0.03; *η*^2^ < 0.001) and maladaptive coping strategy scores (7.1 ± 1.5 for NH-White, 7.0 ± 1.7 for NH-Black, and 7.7 ± 2.1 for Hispanic/Latina participants, *p* < 0.001; *η*^2^ = 0.005). The prevalence of each coping strategy category by race-ethnicity is presented in Figs. 3 and 4.

### Coping as a mediator between both everyday and major differential treatment based on race-ethnicity and sleep duration within racial-ethnic groups

The associations between both everyday and major differential treatment based on race-ethnicity and sleep duration were not statistically significant within the three racial-ethnic groups. This remained unchanged after accounting for coping strategies and covariates. Additionally, coping strategies (adaptive or maladaptive) did not mediate the associations between differential treatment and sleep duration, as the indirect effects were also not significant (Figs. 5A, D, 6A, D, 7A, D).

### Coping as a mediator between both everyday and major differential treatment based on race-ethnicity and poor sleep quality within racial-ethnic groups

#### NH-White women

##### Everyday differential treatment.

While few (3.8%) NH-White women reported everyday differential treatment, the total effect (c path) of everyday differential treatment on poor sleep quality assessed approximately 5–11 years later was statistically significant (unstandardized *beta [b]* = 0.06, standard error [SE] = 0.02, 95% CI [0.02, 0.10], standardized beta [β] = 0.09), and the direct effect (c’ path) remained significant after accounting for coping strategies among NH-White women (*b* = 0.04, SE = 0.02, 95% CI [0.01, 0.08], β = 0.07) (Fig. 5B). The indirect effect of maladaptive coping was significant (indirect effect [a_2_b_2_ path] = 0.02, *SE* = 0.00, 95% CI [0.01, 0.02], β = 0.02). In contrast, adaptive coping did not mediate the relationship between everyday differential treatment and later poor sleep quality among non-Hispanic White women (indirect effect [a_1_b_1_ path] = 0.00, *SE* = 0.00, 95% CI [− 0.00, 0.00], β = 0.00). The overall model explained approximately 9% of the variance in poor sleep quality (*R*^2^ = 0.09).

##### Major differential treatment.

The total effect of major differential treatment on later poor sleep quality among non-Hispanic White women was not statistically significant (*b* = 0.02, SE = 0.03, 95% CI [− 0.03, 0.07], β = 0.03) (Fig. 5E). Maladaptive coping mediated the relationship between major differential treatment and later poor sleep quality (indirect effect = 0.01, SE = 0.00, 95% CI [0.01, 0.02], β = 0.02). In contrast, the pathway through adaptive coping was not significant (indirect effect = 0.00, SE = 0.00, 95% CI [− 0.00, 0.01], β = 0.00). The overall model explained approximately 9% of the variance in poor sleep quality (*R*^2^ = 0.09).

#### NH-Black women

##### Everyday differential treatment.

Among NH-Black women, the total effect (*b* = 0.00, SE = 0.04, 95% CI [− 0.08, 0.08], β = 0.00) and direct effect (*b* = −0.01, SE = 0.04, 95% CI [−0.09, 0.07], β = −0.01) of everyday differential treatment on poor sleep quality assessed approximately 5–11 years later were not statistically significant (Fig. 6B). Maladaptive coping significantly mediated the relationship between everyday differential treatment and later poor sleep quality (indirect effect = 0.01, SE = 0.01, 95% CI [0.00, 0.03], β = 0.02). In contrast, adaptive coping did not mediate this relationship (indirect effect = −0.00, SE = 0.00, 95% CI [− 0.01, 0.00], β = −0.00). The overall model explained approximately 11% of the variance in poor sleep quality (*R*^2^ = 0.11).

##### Major differential treatment.

Neither the direct effect of major differential treatment (*b* = 0.04, SE = 0.03, 95% CI [− 0.02, 0.11], β = 0.06) on later poor sleep quality nor the indirect effects through adaptive (indirect effect = 0.00, *SE* = 0.00, 95% CI [− 0.00, 0.01], β = 0.00) and maladaptive (indirect effect = 0.00, *SE* = 0.00, 95% CI [− 0.00, 0.01], β = 0.01) coping were statistically significant (Fig. 6E). The overall model explained approximately 11% of the variance in poor sleep quality (*R*^2^ = 0.11).

#### Hispanic/Latina women

Among Hispanic/Latina women, the associations between both everyday and major differential treatment and later poor sleep quality were not statistically significant and remained unchanged after accounting for coping strategies and covariates. Additionally, neither adaptive nor maladaptive coping strategies mediated these relationships, as the indirect effects were also not significant (Fig. 7B and E).

### Coping as a mediator between everyday and major differential treatment based on race-ethnicity and insomnia symptoms within racial-ethnic groups

#### NH-White women

##### Everyday differential treatment.

While few (3.8%) NH-White women reported everyday differential treatment, the direct effect of everyday differential treatment on insomnia symptoms assessed approximately 5–11 years later was significant (*b* = 0.24, SE = 0.07, 95% CI [0.10, 0.37]) (Fig. 5C) among NH-White women. Maladaptive coping significantly mediated the relationship between everyday differential treatment and insomnia symptoms, indicating that higher levels of differential treatment were associated with increased maladaptive coping, which in turn was associated with greater insomnia symptoms (indirect effect = 0.03, SE = 0.01, 95% CI [0.02, 0.04]). However, the indirect effect through adaptive coping was not significant (indirect effect = 0.00, SE = 0.00, 95% CI [− 0.00, 0.01]). The overall model explained approximately 5% of the variance in insomnia symptoms (Nagelkerke R^2^ = 0.05).

##### Major differential treatment.

The direct effect of major differential treatment on later insomnia symptoms was not statistically significant (*b* = 0.15, SE = 0.09, 95% CI [− 0.02, 0.32]) (Fig. 5F). Similarly, the mediation pathway through adaptive coping did not reach significance (indirect effect = 0.00, SE = 0.00, 95% CI [− 0.00, 0.01]). However, maladaptive coping significantly mediated the relationship between major differential treatment and later insomnia symptoms (indirect effect = 0.03, SE = 0.01, 95% CI [0.01, 0.04]). The overall model explained approximately 5% of the variance in insomnia symptoms (Nagelkerke R^2^ = 0.05).

#### NH-Black women

##### Everyday differential treatment.

Among non-Hispanic Black women, the direct effect of everyday differential treatment on later insomnia symptoms was not statistically significant (*b* = 0.05, SE = 0.13, 95% CI [− 0.20, 0.31]) (Fig. 6C). Maladaptive coping significantly mediated the relationship between everyday differential treatment and later insomnia symptoms (indirect effect = 0.04, SE = 0.02, 95% CI [0.01, 0.07]). In contrast, adaptive coping did not mediate the relationship (indirect effect= −0.00, SE = 0.00, 95% CI [− 0.01, 0.01]). The overall model explained approximately 8% of the variance in insomnia symptoms (Nagelkerke R^2^ = 0.08).

##### Major differential treatment.

Neither the direct effect of major differential treatment (*b* = 0.06, SE = 0.11, 95% CI [− 0.15, 0.26]) on later insomnia symptoms nor the indirect effects through adaptive (indirect effect = 0.00, SE = 0.00, 95% CI [− 0.01, 0.01]) and maladaptive (indirect effect = 0.01, SE = 0.01, 95% CI [− 0.01, 0.03]) coping reached statistical significance (Fig. 6F). The overall model explained approximately 8% of the variance in insomnia symptoms (Nagelkerke R^2^ = 0.08).

#### Hispanic/Latina women

Among Hispanic/Latina women, the associations between both everyday and major differential treatment and insomnia symptoms assessed approximately 5–11 years later were not statistically significant and remained unchanged after accounting for coping strategies and covariates. Additionally, neither adaptive nor maladaptive coping strategies mediated these relationships, as the indirect effects were also not significant (Fig. 7).

### Secondary analyses: moderated mediation by educational attainment and household income

We examined whether educational attainment and household income moderated the indirect effect of everyday and major differential treatment based on race-ethnicity on later sleep health through adaptive and maladaptive coping. Among NH-White women, household income significantly moderated the effects of both everyday and major differential treatment on poor sleep quality and insomnia symptoms, but not sleep duration, through maladaptive coping, particularly when comparing the $50,000–$100,000 income group to those earning less than $50,000 (see Supplemental Tables 1 and 2). For everyday differential treatment, the index of moderated mediation (IMM) indicated that the mediation effect of maladaptive coping is weaker in the $50,000–$100,000 group compared with the lower-income (< $50,000) group (IMM_poor sleep quality_ = −0.01, SE = 0.01, 95% CI [− 0.02, − 0.00], R^2^ = 0.09; IMM_insomnia symptoms_ = −0.03, SE = 0.01, 95% CI [− 0.05, − 0.00], Nagelkerke R^2^ = 0.05). Similarly, for major differential treatment, the IMM for poor sleep quality (IMM = −0.02, SE = 0.01, 95% CI [− 0.03, − 0.00], R^2^ = 0.09) and insomnia symptoms (IMM = −0.04, SE = 0.02, 95% CI [− 0.07, − 0.01], Nagelkerke R^2^ = 0.05) suggested that the impact of differential treatment on sleep through maladaptive coping is lower for the $50,000–$100,000 compared with the < $50,000 income group. This pattern was not observed for those earning $100,000 or more, where the moderated mediation effects were not significant, indicating that the relationship between differential treatment and sleep through maladaptive coping is particularly pronounced in the lower-income group. Additionally, educational attainment did not moderate the mediation between differential treatment and sleep through coping among NH-White women. In contrast, among both NH-Black and Hispanic/Latina women, neither household income nor educational attainment significantly moderated the indirect effects of everyday or major differential treatment on sleep health through either adaptive or maladaptive coping—whether considering sleep duration, poor sleep quality, or insomnia symptoms (see Supplemental Tables 3–6).

## Discussion

In this large cohort of middle-to-older-aged NH-White, NH-Black/African American, and Hispanic/Latina women in the United States, we found that among NH-Black/African American women, maladaptive coping mediated the relationship between everyday differential treatment based on race-ethnicity and poor sleep quality and insomnia symptoms 5–11 years later, while adaptive coping did not. Contrary to our hypothesis, neither coping strategy significantly mediated the relationship between major differential treatment based on race-ethnicity and later sleep health among NH-Black/African American women. Among Hispanic/Latina women, neither maladaptive nor adaptive coping strategies significantly mediated the relationship between everyday or major differential treatment based on race-ethnicity and sleep 5–11 years later. Among NH-White women, despite the low prevalence of reported differential treatment, everyday and major differential treatment were associated with maladaptive coping, which in turn was associated with poor sleep quality and insomnia symptoms 5–11 years later; however, adaptive coping did not mediate relationships. These findings extend prior Sister Study analyses, which established direct associations between differential treatment and multiple sleep dimensions (Gaston et al., 2020), by evaluating coping strategies as potential mediators of these relationships.

Prior studies suggest that coping strategies influence how differential treatment affects sleep (Hoggard & Hill, 2018; Majeno et al., 2022), and that the use of maladaptive coping strategies is higher among individuals with insomnia symptoms (Li et al., 2021; Otsuka et al., 2022). Consistent with these findings, our results show that maladaptive coping functions as a behavioral pathway through which differential treatment based on race-ethnicity contributes to poorer sleep health, particularly insomnia symptoms and poor sleep quality. One study in the United States found that adequate sleep facilitated the use of active coping strategies, such as problem-solving and seeking peer support, which in turn promoted overall well-being (Wang & Yip, 2020). In contrast, among Latinx adults, coping strategies modified the relationship between differential treatment and sleep, with those using a combination of passive and active strategies experiencing poorer sleep quality under everyday differential treatment, while those relying on active coping alone exhibited greater sleep disturbances under major lifetime differential treatment (Majeno et al., 2022). Our null findings for adaptive coping as a mediator are consistent with this observation, suggesting that adaptive coping strategies do not uniformly protect against the effects of differential treatment and may, in some cases, heighten stress responses depending on the context and persistence of the stressor. This may reflect the limited effectiveness of individual-level coping strategies in the context of chronic and pervasive stressors such as differential treatment, as well as the possibility that the coping strategies assessed do not capture culturally relevant or collective forms of coping. Additionally, research among Japanese workers indicated that those experiencing insomnia symptoms were more likely to engage in maladaptive coping strategies compared with workers without sleep difficulties (Otsuka et al., 2022), which aligns with our observation that higher maladaptive coping scores were associated with greater insomnia symptoms several years later, supporting the idea that maladaptive coping may perpetuate sleep-related distress over time.

Furthermore, prior Sister Study analyses found that both everyday and major differential treatment based on raceethnicity were directly associated with multiple dimensions of sleep, including short sleep duration, insomnia symptoms, and poor sleep quality (Gaston et al., 2020). Our study extends this work by demonstrating that coping, particularly maladaptive coping, partially explains these associations and provides evidence for a mediating pathway between differential treatment and sleep health. Maladaptive coping strategies may trigger physiological stress responses, disrupting the transition from wakefulness to sleep and interfere with the body’s ability to achieve restorative sleep stages (Bell et al., 2019; Thomas et al., 2006; Tomfohr et al., 2010). Differential treatment related psychological distress, exacerbated by ineffective coping, could create a feedback loop amplifying the impact on sleep health, leading to insomnia symptoms and sleep disturbances (Hicken et al., 2013; Todorova et al., 2010). The lack of mediation through adaptive coping strategies may indicate that certain positive coping mechanisms do not provide the same level of protection against the negative effects of perceived differential treatment on sleep health.

The differential impact of coping strategies on various dimensions of sleep health years later underscores the need for a more nuanced understanding of their role in the sleep process. While coping strategies may influence poor sleep quality and insomnia symptoms, their impact on sleep duration may be mediated by socio-environmental factors. The EA model suggests that environments can either constrain or facilitate coping resources, which in turn shape sleep health (Mezuk et al., 2013). We use the EA model as a guiding framework to contextualize the role of coping, conceptualizing differential treatment as a stressor, coping strategies as stress responses, and sleep health as an outcome. Our analyses focus on this central pathway within the EA framework, while acknowledging that other domains, such as, early life experiences and neighborhood condition also shape sleep health. Research indicates that these factors, including neighborhood (e.g., safety, cohesion), household environment (e.g., temperature, light), and socioeconomic conditions (e.g., occupation status) contribute to racial differences observed in sleep duration (Alhasan et al., 2020; Gaston et al., 2024; Jackson et al., 2013; Johnson et al., 2021). Future research should consider these multifaceted influences to elucidate the complex mechanisms underlying the relationship between coping strategies and sleep duration.

The differences observed across racial-ethnic groups may be attributed to contextual factors, such as historical and socio-cultural influences. For instance, among NH-White women, the association between perceived race-based differential treatment and maladaptive coping may reflect a response to relatively infrequent experiences with differential treatment based on race-ethnicity (4% everyday differential treatment and 2% major differential treatment), making maladaptive strategies more salient when such experiences occur. Additionally, NH-White participants in this cohort include individuals of Middle Eastern descent, who may experience distinct forms of differential treatment (e.g., anti-Muslim differential treatment), and prior analyses in this cohort have suggested stronger associations between differential treatment and sleep outcomes in this subgroup compared to NH-White women of European descent (Gaston et al., 2020). Importantly, these findings should be interpreted in the context of the relatively low prevalence of reported differential treatment based on race-ethnicity among NH-White women and should not be taken to indicate that differential treatment is a primary driver of sleep health in this group. In contrast, NH-Black/African American women, who often face more pervasive and systemic differential treatment, may rely on maladaptive coping strategies (e.g., denial, venting, behavioral disengagement) as a means of navigating persistent adversity (K. J. Homan 2024a, b; Ward et al., 2019). Further, cultural influences, resilience, and communal coping mechanisms might contribute to a broader range of coping strategies that go beyond the strategies measured in our study (e.g., community-based support networks) (Christophe et al., 2022; Jacob et al., 2023; Jones et al., 2020; West et al., 2010). Moreover, a multifaceted perspective, which emphasizes how overlapping forces, such as differential treatment based on race, sex, and nationality, interact to shape experiences, suggests that the experience of differential treatment is complex and multidimensional (Bowleg, 2021; Lewis et al., 2017). These intersecting forces may complicate the relationship between differential treatment and sleep, particularly among the Hispanic/Latina group, as the sample included women residing both in Puerto Rico and on the U.S. mainland. Differences in geographic location may contribute to varying experiences of differential treatment, coping responses, and sleep environments. Women residing in Puerto Rico may have distinct environmental exposures compared with those living on mainland United States, potentially influencing their likelihood of experiencing differential treatment and the ways in which the group copes with stressors. Among Puerto Ricans, darker skin tone has been associated with greater exposure to major differential treatment, which contributed to increased physiological dysregulation, including higher allostatic load, a measure of cumulative biological stress (Cuevas et al., 2021). These findings highlight how skin tone influences social experiences and health outcomes within Hispanic/Latina populations. This geographic, cultural, and phenotypic variation may partially explain why expected associations were not observed in this study. Future research should account for regional differences and consider Hispanic/Latina women from other heritage groups when examining the relationship between differential treatment, coping, and sleep health among Hispanic/Latina women.

Our findings underscore the importance of considering both the associations between perceived differential treatment, coping, and sleep health, as well as the prevalence of differential treatment exposure when identifying targets for intervention. The higher prevalence of differential treatment based on race-ethnicity reported by NH-Black and Hispanic/Latina women in this relatively high SES cohort has public health relevance and can inform public health intervention strategies. As noted by Ward et al. (2019), understanding the prevalence is essential because it contextualizes the scale and potential impact of differential treatment experiences on health outcomes, particularly when such exposure is widespread. In this context, our findings regarding the prevalence of differential treatment (79% everyday differential treatment and 57% major differential treatment among NH-Black women; 29.7% everyday differential treatment and 14.3% major differential treatment among Hispanic/Latina women) are especially notable, as they suggest that a large portion of NH-Black and Hispanic/Latina women, even those who have high educational attainment and financial resources such as those included in this cohort are routinely encountering stressors that are known to adversely impact health. Although some of the indirect effects we observed were modest, even small effects can have meaningful public health relevance when exposures like differential treatment based on race-ethnicity are both common and persistent (Bécares & Zhang, 2018; Ward et al., 2019; Williams et al., 2019). Prior work has shown that small but consistent mediation effects can provide valuable mechanistic insight and, when accumulated over time and across populations, contribute to substantial health disparities (Fuller-Rowell et al., 2017; Hisler & Brenner, 2019; Mead et al., 2023; Tamura et al., 2022). Taken together, such data on both the differential treatment prevalence and impact of differential treatment can inform public health policy and practice by guiding the creation of culturally tailored mental health resources and enhancing the prioritization and allocation of resources to support the most affected groups.

Our study has several limitations. First, the assessment of coping strategies was not exhaustive and may oversimplify a complex and context-dependent construct. We operationalized coping into ‘adaptive’ and ‘maladaptive’ categories using a nine-item measure, which does not capture the full range of coping responses. Certain strategies (e.g., acceptance, disengagement) may function adaptively in some situations and maladaptively in others, depending on cultural norms and the chronicity of stressors (Waugh et al., 2020). In addition, our measure did not include culturally salient strategies such as spiritual/religious practices, mindfulness/meditation practices, collective/community-based coping, activism, or substance-related coping (Caro et al., 2023; Jacob et al., 2023). Even though capturing these strategies is important, they were not available in the Sister Study data. As such, our findings should be interpreted as applying only to the subset of coping strategies assessed, and future research should examine more comprehensive and culturally relevant measures of coping. Second, relying on self-reported differential treatment and sleep data may introduce bias (e.g., misclassification, recall bias) (Gaston & Jackson, 2022; Jackson et al., 2018). A validation study investigating the agreement between self-reported and objectively measured sleep revealed that self-reported sleep duration was consistently overestimated across all racial-ethnic groups compared with objective sleep duration, which indicates that our observation is not solely attributable to measurement bias (Jackson et al., 2018). Third, the smaller sample sizes for NH-Black/African American and Hispanic/Latina women limit the precision of our estimates. In particular, the number of participants residing in Puerto Rico was too small to permit separate subgroup analyses, as was done in Gaston et al. (2020). This limited our ability to fully examine potential geographic differences in differential treatment, coping, and sleep within the Hispanic/Latina group. As a result, the findings for the Hispanic/Latina women did not capture contextual factors that could help explain the absence of significant associations observed in this study. Third, the generalizability of the findings to the broader population is limited due to the non-nationally representative sample of the Sister Study population and the lack of data on other racial-ethnic groups. Lastly, since analyses were hypothesis-driven and informed by prior literature, no formal correction for multiple comparisons was applied. Nonetheless, the possibility of Type I error cannot be ruled out because multiple mediation models were estimated across types of differential treatment, coping strategies, and sleep outcomes. Findings should be interpreted with this consideration in mind.

Despite the limitations, our study has important strengths and has notable clinical and public health implications. For instance, we investigated both everyday and major differential treatment based on race-ethnicity, as well as multiple sleep dimensions using data from a varied group of women in a large cohort study. This approach allowed for separate mediation analyses by race-ethnicity while accounting for many potential confounders. The identification of maladaptive coping as a mediator suggests that individual-level interventions targeting coping skills may be valuable in addressing the adverse impact of differential treatment on sleep health. Culturally tailored interventions that consider the unique experiences of each racial-ethnic group may be particularly effective. Routine clinical assessments that include evaluations of coping strategies, particularly in individuals reporting differential treatment experiences, can facilitate early identification and intervention to improve sleep health, as well as overall mental and physical health outcomes. Further, collaboration between sleep medicine specialists, mental health practitioners, and primary care providers is essential to provide an integrated care approach that addresses both coping strategies and sleep health. While strengthening coping resources is critical, interventions must also address broader environmental and community factors that shape stress exposure and resilience. Initiatives aimed at fostering welcoming and supportive environments are necessary alongside coping-based interventions to ensure long-term improvements in sleep and well-being for all individuals. Moreover, public health efforts raising awareness about the importance of sleep health and underlying psychosocial processes, including differential treatment, that contribute to sleep disparities, are crucial for driving improvements in sleep health.

In conclusion, maladaptive coping may serve as a mechanism through which perceived differential treatment based on race-ethnicity disrupts sleep health. Further research is warranted to investigate additional contextual factors that may influence the relationship between differential treatment, coping strategies, and sleep health, such as support networks and cultural norms. Further research is also warranted to examine the bidirectional associations between differential treatment, coping, and sleep. Furthermore, qualitative research approaches may offer a deeper understanding of individual experiences of differential treatment and awareness of coping strategies within varied cultural contexts.

## Supplementary Material

supplemental material

The online version contains supplementary material available at https://doi.org/10.1007/s10865-026-00710-1.

## Figures and Tables

**Fig. 1:**
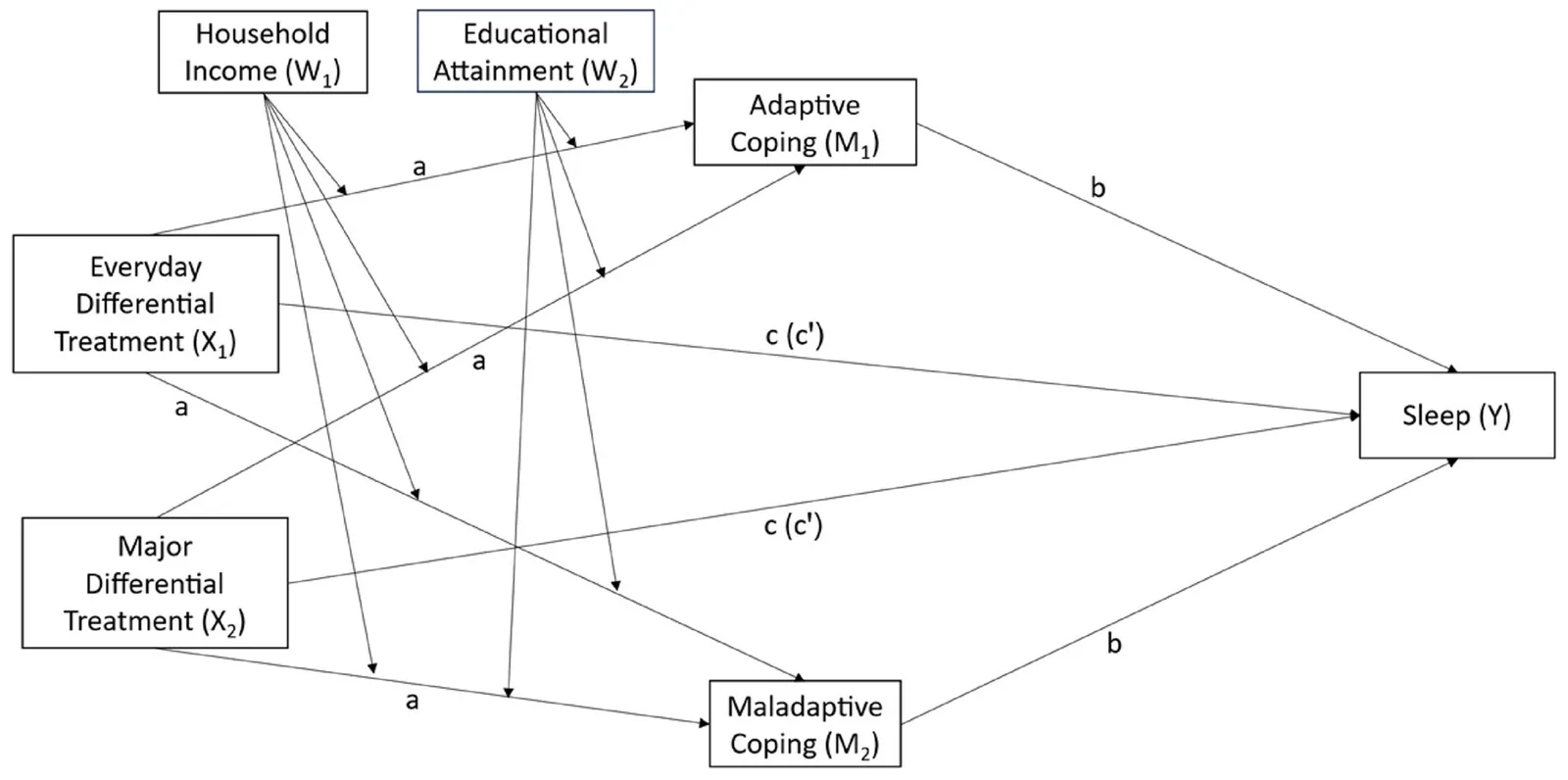
A conceptual mediation model

**Fig. 2:**
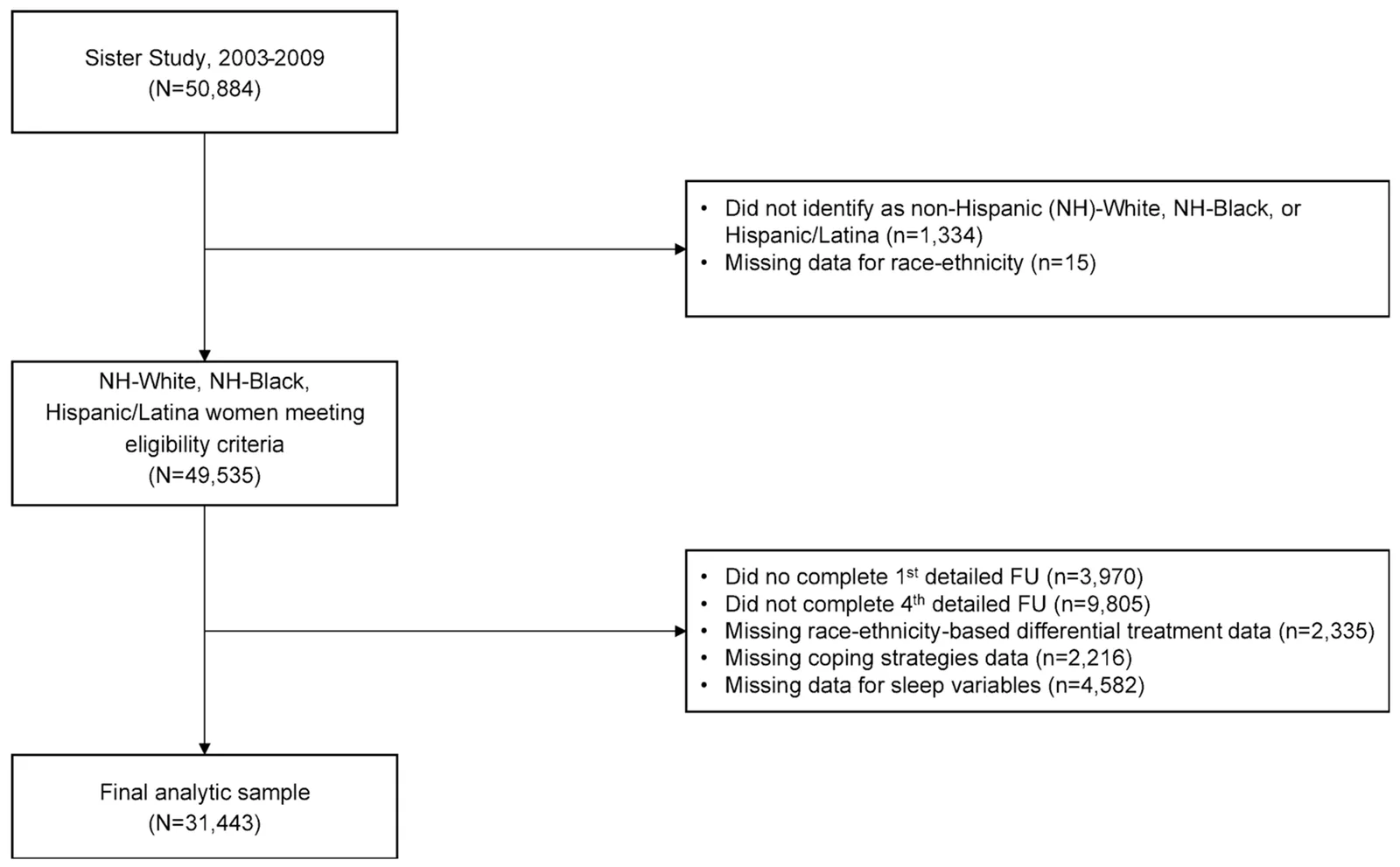
Composition of analytic sample. *Note*. Exclusion criteria were applied simultaneously; therefore, exclusion counts are not mutually exclusive. Some participants met multiple criteria; the final analytic sample reflects unique exclusions (N = 31,443)

**Fig. 3:**
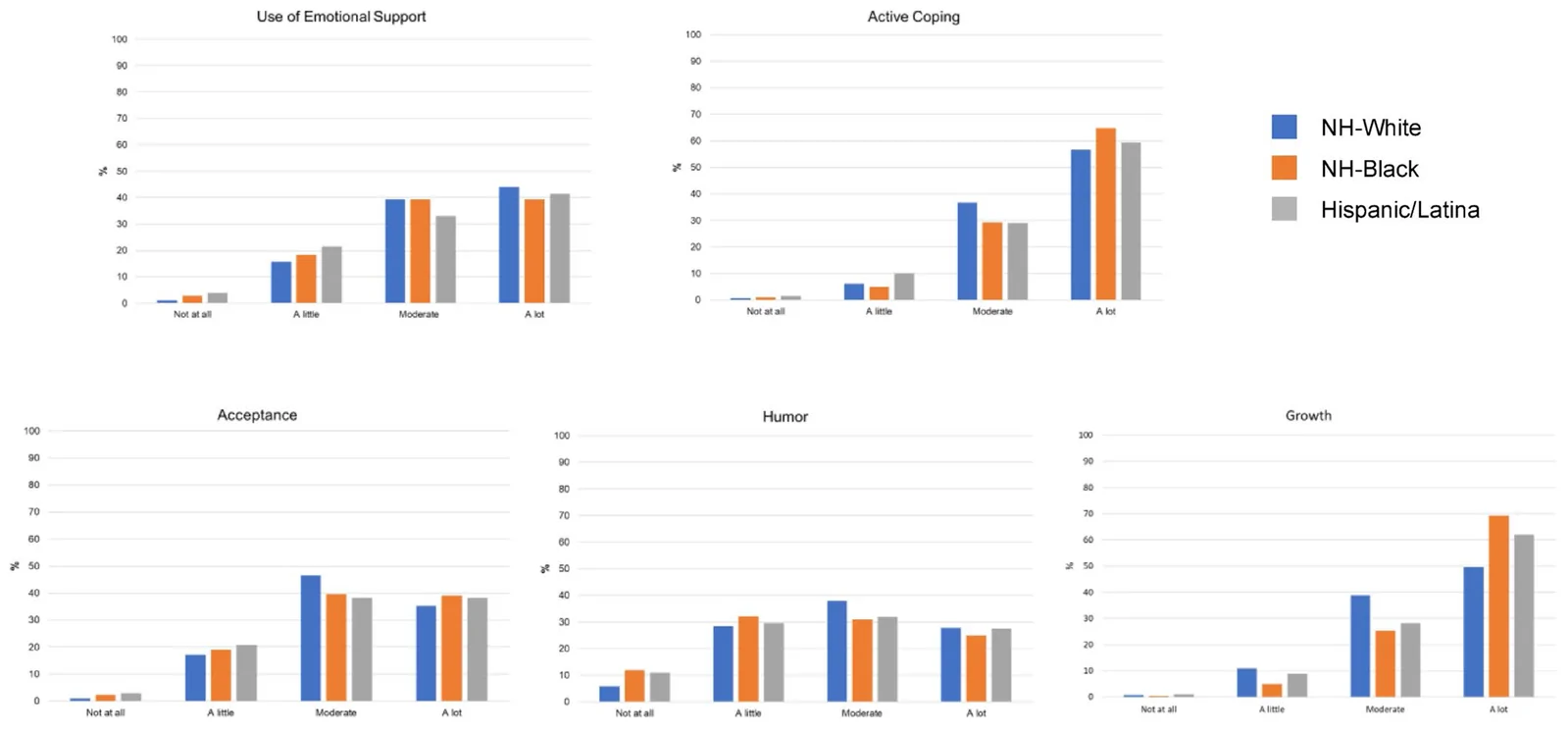
Healthy/adaptive coping strategies by race-ethnicity

**Fig. 4:**
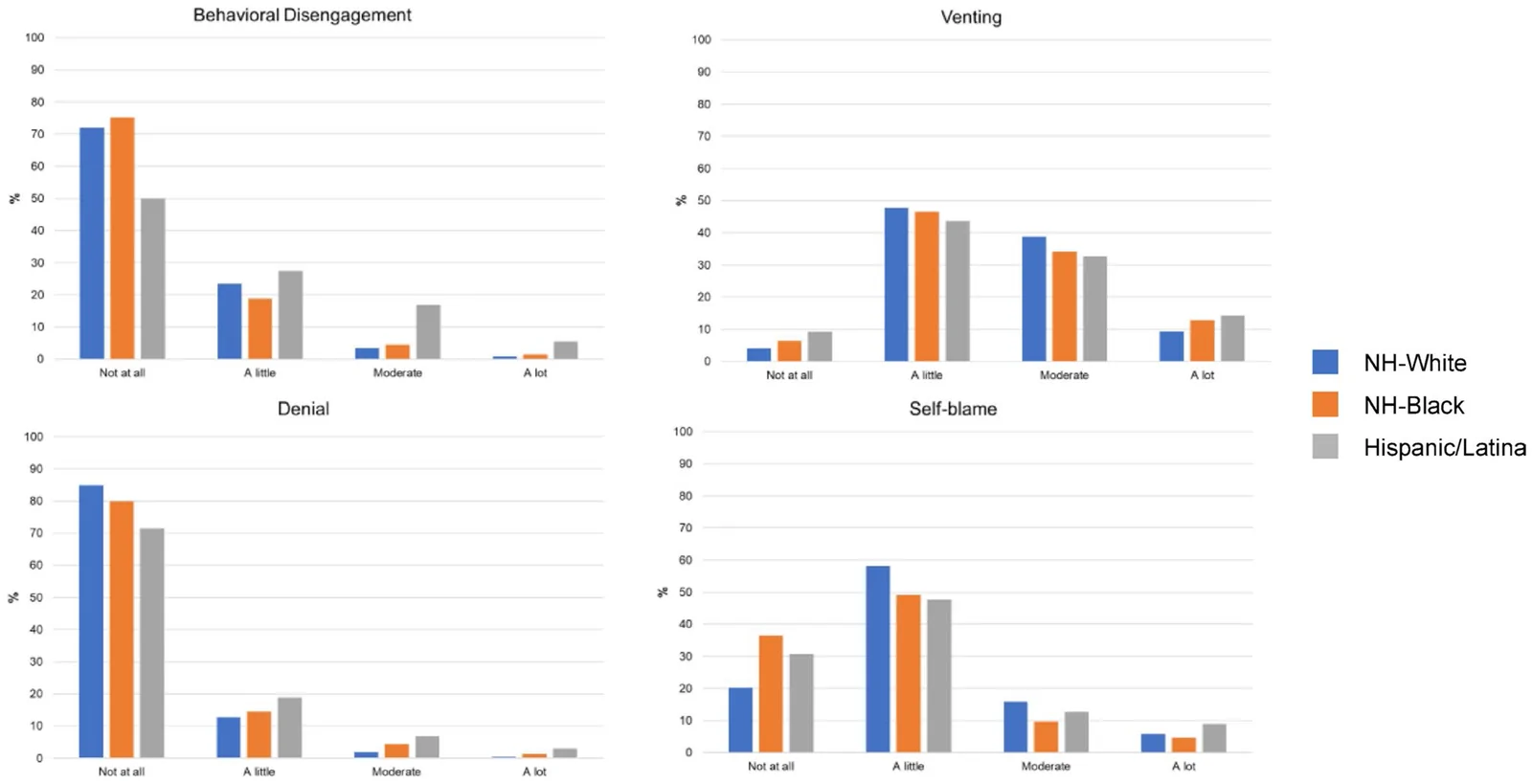
Maladaptive coping strategies by race-ethnicity

**Fig. 5:**
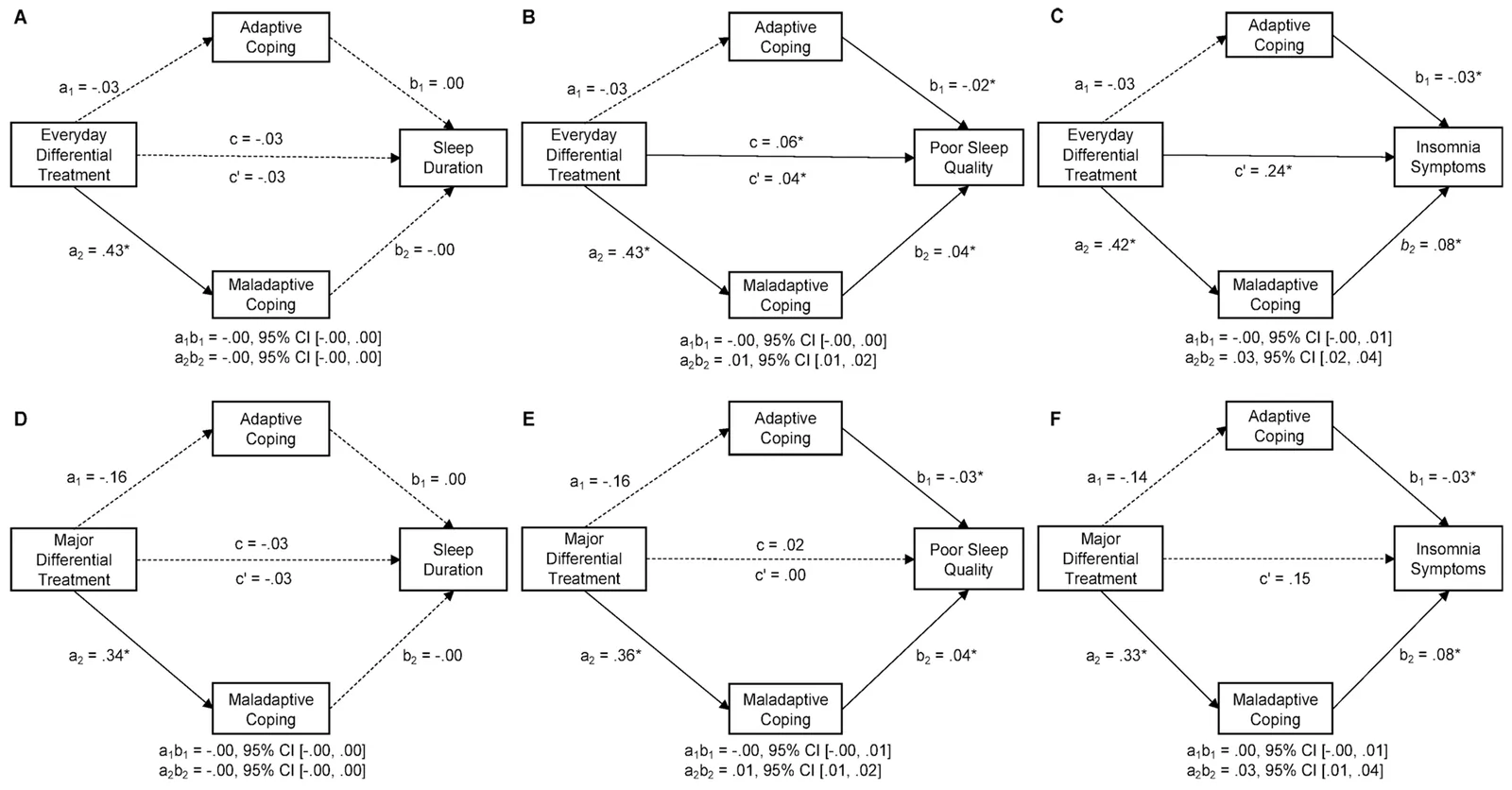
Parallel mediation model illustrating the association between race-ethnicity-based differential treatment and sleep dimensions, simultaneously through adaptive and maladaptive coping among non-Hispanic White women. Total effect could not be estimated with dichotomous insomnia symptoms variable. Models adjusted for baseline age, marital status, employment status, educational attainment, household income, and body mass index, depressive symptoms at 1^st^ follow-up, and general health status and sleep medication use at 4^th^ follow-up. *p < 0.05; solid line = significant association

**Fig. 6:**
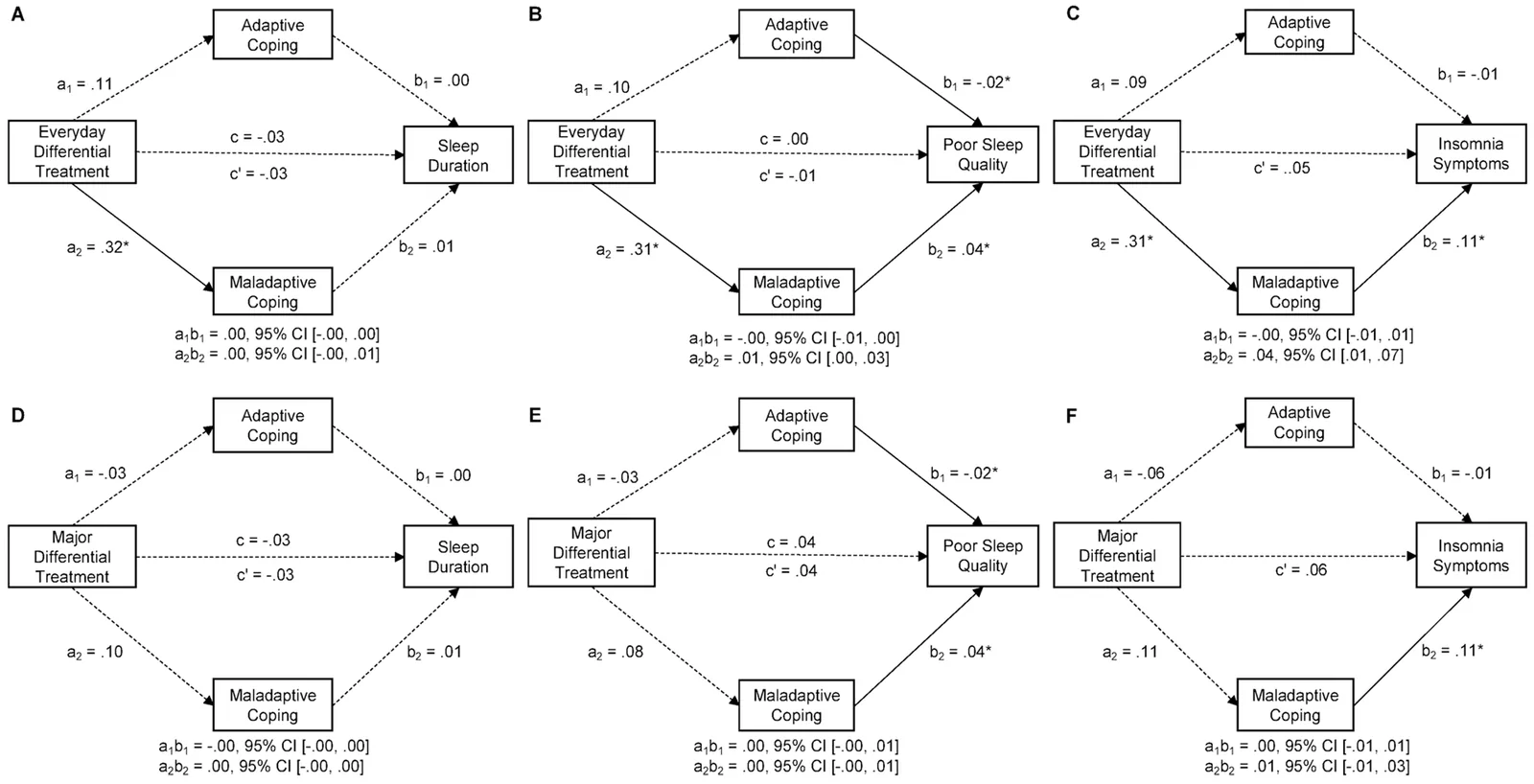
Parallel mediation model illustrating the association between race-ethnicity-based differential treatment and sleep dimensions, simultaneously through adaptive and maladaptive coping among non-Hispanic Black women. Total effect could not be estimated with dichotomous insomnia symptoms variable. Models adjusted for baseline age, marital status, employment status, educational attainment, household income, and body mass index, depressive symptoms at 1^st^ follow-up, and general health status and sleep medication use at 4^th^ follow-up. *p < 0.05; solid line = significant association

**Fig. 7:**
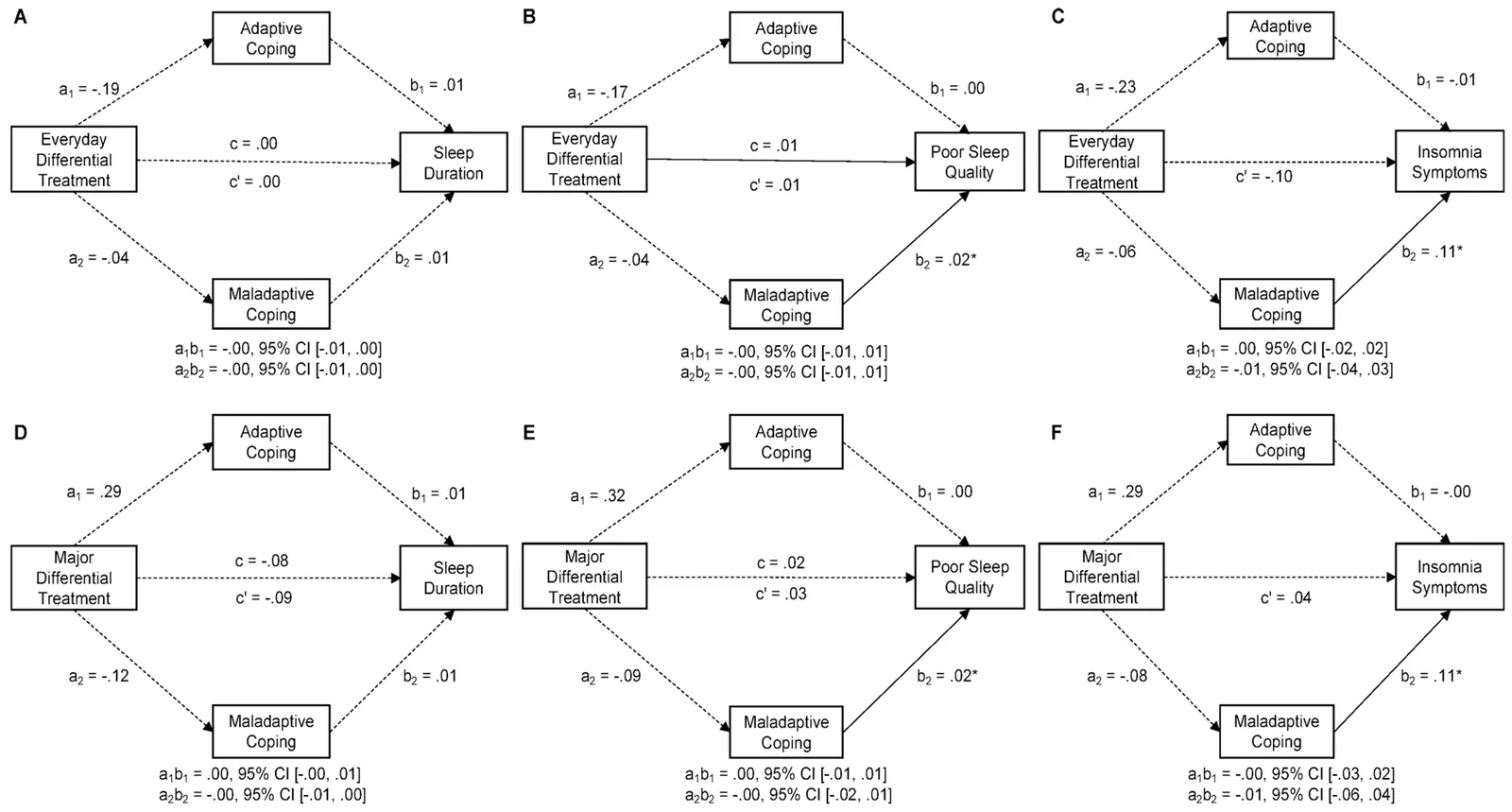
Parallel mediation model illustrating the association between race-ethnicity-based differential treatment and sleep dimensions, simultaneously through adaptive and maladaptive coping among Hispanic women. Total effect could not be estimated with dichotomous insomnia symptoms variable. Models adjusted for baseline age, marital status, employment status, educational attainment, household income, and body mass index, depressive symptoms at 1^st^ follow-up, and general health status and sleep medication use at 4^th^ follow-up. *p < 0.05; solid line = significant association; CI, confidence interval

**Table 1:** Sociodemographic characteristics, health behaviors and clinical characteristics, coping measures, and experiences of differential treatment based on race-ethnicity among included participants, Sister Study (2003–2019)

	Total *N* = 31,443	NH-White *n* = 28,619 (91.0%)	NH-Black *n* = 1,838 (5.8%)	Hispanic/Latina *n* = 986 (3.1%)	*p*-value
*Sociodemographic characteristics*					
Mean age ± SD (years)	55.5 ± 8.6	55.7 ± 8.6	54.2 ± 8.0	52.5 ± 8.6	
Age category					
35–64 years	84.0	83.3	91.3	91.7	< 0.001
≥ 65 years	16.0	16.7	8.7	8.3	
Educational attainment					
≤ High school	12.7	12.9	7.1	17.1	< 0.001
Some college or technical degree	31.2	31.3	30.3	31.7	
≥ College (Bachelor’s or greater)	56.0	55.8	62.7	51.1	
Employment status					
Unemployed	32.2	32.7	24.1	32.2	< 0.001
Currently employed	67.8	67.3	75.9	67.8	
Annual household income					
< $20,000 to $49,999	21.1	20.2	25.4	38.0	< 0.001
$50,000 to $99,999	41.9	41.9	45.1	35.7	
≥ $100,000	37.0	37.9	29.5	26.3	
Marital status					
Married/living as married	77.6	79.2	55.0	71.7	< 0.001
Single/never married	5.0	4.4	12.6	6.5	
Divorced/separated/widowed	17.5	16.3	32.4	21.8	
*Health behaviors*					
Habitual sleep duration					
Short sleep (< 7 h)	11.4	10.0	29.8	16.2	< 0.001
Recommended sleep (7–9 h)	73.9	75.1	58.4	67.3	
Long sleep (> 9 h)	14.7	14.9	11.8	16.6	
Poor sleep quality					
None to slight	75.5	75.7	73.1	75.0	0.001
Mild	14.8	14.9	14.8	13.7	
Moderate/severe	9.7	9.5	12.2	11.3	
Insomnia symptoms (yes)	43.4	43.8	39.9	38.3	< 0.001
Sleep medication use category					
Not in the past month	65.3	64.4	77.0	71.1	< 0.001
< 1 time/week	9.6	9.8	6.3	8.4	
1–2 times/week	6.4	6.5	6.0	6.6	
≥ 3 times/week	18.7	19.4	10.7	13.9	
*Clinical characteristics*					
Health status (past 12 months)					
Poor/fair	7.4	6.9	9.7	13.5	< 0.001
Good	24.5	23.7	35.2	28.2	
Very good/excellent	68.1	69.3	55.1	58.3	
Body mass index (BMI)					
Underweight/recommended (BMI < 24.9 kg/m^2^)	42.7	44.5	18.2	35.5	< 0.001
Overweight (25 kg/m^2^ ≤ BMI ≤ 29.9 kg/m^2^)	31.8	31.5	34.4	35.6	
Obesity (BMI ≥ 30 kg/m^2^)	25.5	24.0	47.5	28.8	
*Coping and mental health*					
Adaptive coping style, mean ± SD	16.2 ± 2.4	16.2 ± 2.4	16.2 ± 2.3	16.0 ± 2.6	0.03
Maladaptive coping style, mean ± SD	7.1 ± 1.6	7.1 ± 1.5	7.0 ± 1.7	7.7 ± 2.1	< 0.001
Depressive symptoms (Yes) ^a^	1.3	1.2	1.3	2.8	< 0.001
*Perceived differential treatment* ^b^					
Everyday differential treatment (Yes)	9.0	3.8	78.6	29.7	< 0.001
Major differential treatment (Yes)	5.8	2.2	57.1	14.3	< 0.001

NH (non-Hispanic); SD (standard deviation)

a Depressive symptoms were measured using the 10-item Center for Epidemiological Studies-Depression (CES-D-10), with scores of 10 or higher indicating depressive symptoms

b Everyday differential treatment was recorded as “yes” if participants self-reported ever experiencing being treated unfairly in receiving service at a store or restaurant; being treated as less intelligent, worthy or honest than others; and experiencing people acting as if they are afraid of you due to your race or ethnicity. Major differential treatment was recorded as “yes” if participants self-reported ever experiencing being treated unfairly in home renting, buying or mortgage lending; treated unfairly in being stopped, searched, or threatened by police; and treated unfairly in job hiring, promotion, or firing due to your race or ethnicity

Sociodemographic characteristics and BMI were assessed at baseline. Perceived differential treatment based on race and ethnicity, coping style, and depressive symptoms were assessed at the first detailed follow-up. Sleep dimensions, sleep medication use, and health status were assessed at fourth detailed follow-up

## Data Availability

Data, including anonymized data for replication, are available as described on the Sister Study website (https://sisterstudy.niehs.nih.gov/English/coll-data.htm). Data use is restricted to replication, only. New analyses must be approved after a proposal process. Code can be requested from the corresponding author.
