# Supplementary material for: Maladaptive coping strategies mediate the relationship between perceived differential treatment and sleep health among women
Source: J Behav Med. 2026 Sep 7;49(6):1043–60. doi: 10.1007/s10865-026-00710-1 (PMC13631875; doi:10.1007/s10865-026-00710-1)
Supplement: Supplementary file 1 [file 10865_2026_710_MOESM1_ESM.docx]

| Supplemental Table 1. Conditional indirect effect of everyday differential treatment on sleep dimensions through adaptive and maladaptive coping among non-Hispanic White women. | | | | | |
| --- | --- | --- | --- | --- | --- |
|  | | Conditional Indirect effect | | | |
| Mediator | Moderation effect on a path | *b* | SE | 95% Confidence Interval | |
| **Sleep duration** | | | | | |
| Different levels of Educational Attainment | | | | | |
| Adaptive Coping | ≤High school | .00 | .00 | -.00 | .00 |
|  | Some college or technical degree | .00 | .00 | -.00 | .00 |
|  | ≥college (bachelor’s or greater) | -.00 | .00 | -.00 | .00 |
| Index of moderated mediation: some college vs. ≤High school | | -.00 | .00 | -.00 | .00 |
| Index of moderated mediation: ≥college vs. ≤High school | | -.00 | .00 | -.00 | .00 |
| Maladaptive coping | ≤High school | -.00 | .00 | -.00 | .00 |
|  | Some college or technical degree | -.00 | .00 | -.00 | .00 |
|  | ≥college (bachelor’s or greater) | -.00 | .00 | -.00 | .00 |
| Index of moderated mediation: some college vs. ≤High school | | .00 | .00 | -.00 | .00 |
| Index of moderated mediation: ≥college vs. ≤High school | | .00 | .00 | -.00 | .00 |
|  | | | | | |
| Different levels of Household Income | | | | | |
| Adaptive Coping | <$50,000 | -.00 | .00 | -.00 | .00 |
|  | $50,000 - <$100,000 | -.00 | .00 | -.00 | .00 |
|  | ≥$100,000 | .00 | .00 | -.00 | .00 |
| Index of moderated mediation: $50,000 - <$100,000 vs. <$50,000 | | -.00 | .00 | -.00 | .00 |
| Index of moderated mediation: ≥$100,000 vs. <$50,000 | | .00 | .00 | -.00 | .00 |
| Maladaptive coping | <$50,000 | -.00 | .00 | -.00 | .00 |
|  | $50,000 - <$100,000 | -.00 | .00 | -.00 | .00 |
|  | ≥$100,000 | -.00 | .00 | -.00 | .00 |
| Index of moderated mediation: $50,000 - <$100,000 vs. <$50,000 | | .00 | .00 | -.00 | .00 |
| Index of moderated mediation: ≥$100,000 vs. <$50,000 | | .00 | .00 | -.00 | .00 |
| **Poor Sleep Quality** | | | | | |
| Different levels of Educational Attainment | | | | | |
| Adaptive Coping | ≤High school | -.00 | .00 | -.01 | .01 |
|  | Some college or technical degree | -.00 | .00 | -.01 | .00 |
|  | ≥college (bachelor’s or greater) | .00 | .00 | -.00 | .01 |
| Index of moderated mediation: some college vs. ≤High school | | .00 | .01 | -.01 | .01 |
| Index of moderated mediation: ≥college vs. ≤High school | | .01 | .01 | -.01 | .02 |
| Maladaptive coping | ≤High school | .02 | .01 | .00 | .04 |
|  | Some college or technical degree | .02 | .00 | .01 | .03 |
|  | ≥college (bachelor’s or greater) | .01 | .00 | .01 | .02 |
| Index of moderated mediation: some college vs. ≤High school | | .00 | .01 | -.02 | .02 |
| Index of moderated mediation: ≥college vs. ≤High school | | -.01 | .01 | -.02 | .01 |
|  | | | | | |
| Different levels of Household Income | | | | | |
| Adaptive Coping | <$50,000 | .00 | .00 | -.00 | .01 |
|  | $50,000 - <$100,000 | .00 | .00 | -.00 | .01 |
|  | ≥$100,000 | -.00 | .00 | -.01 | .00 |
| Index of moderated mediation: $50,000 - <$100,000 vs. <$50,000 | | .00 | .00 | -.01 | .01 |
| Index of moderated mediation: ≥$100,000 vs. <$50,000 | | -.00 | .00 | -.01 | .00 |
| Maladaptive coping | <$50,000 | .02 | .00 | .01 | .03 |
|  | $50,000 - <$100,000 | .01 | .00 | .00 | .02 |
|  | ≥$100,000 | .02 | .00 | .01 | .02 |
| Index of moderated mediation: $50,000 - <$100,000 vs. <$50,000 | | -.01 | .01 | -.02 | -.00 |
| Index of moderated mediation: ≥$100,000 vs. <$50,000 | | -.01 | .01 | -.02 | .00 |
| **Insomnia Symptoms** | | | | | |
| Different levels of Educational Attainment | | | | | |
| Adaptive Coping | ≤High school | -.01 | .01 | -.02 | .01 |
|  | Some college or technical degree | -.00 | .00 | -.01 | .01 |
|  | ≥college (bachelor’s or greater) | .00 | .00 | -.00 | .01 |
| Index of moderated mediation: some college vs. ≤High school | | .00 | .01 | -.01 | .02 |
| Index of moderated mediation: ≥college vs. ≤High school | | .01 | .01 | -.01 | .02 |
| Maladaptive coping | ≤High school | .04 | .02 | .01 | .08 |
|  | Some college or technical degree | .04 | .01 | .02 | .06 |
|  | ≥college (bachelor’s or greater) | .03 | .01 | .02 | .04 |
| Index of moderated mediation: some college vs. ≤High school | | -.00 | .02 | -.04 | .04 |
| Index of moderated mediation: ≥college vs. ≤High school | | -.02 | .02 | -.06 | .02 |
|  | | | | | |
| Different levels of Household Income | | | | | |
| Adaptive Coping | <$50,000 | .00 | .00 | -.01 | .01 |
|  | $50,000 - <$100,000 | .00 | .00 | -.00 | .01 |
|  | ≥$100,000 | -.00 | .00 | -.01 | .00 |
| Index of moderated mediation: $50,000 - <$100,000 vs. <$50,000 | | .00 | .01 | -.01 | .01 |
| Index of moderated mediation: ≥$100,000 vs. <$50,000 | | -.01 | .01 | -.02 | .00 |
| Maladaptive coping | <$50,000 | .04 | .01 | .03 | .07 |
|  | $50,000 - <$100,000 | .02 | .01 | .01 | .04 |
|  | ≥$100,000 | .03 | .01 | .02 | .05 |
| Index of moderated mediation: $50,000 - <$100,000 vs. <$50,000 | | -.03 | .01 | -.05 | -.00 |
| Index of moderated mediation: ≥$100,000 vs. <$50,000 | | -.01 | .01 | -.04 | .01 |

Note. All models adjusted for baseline age, marital status, employment status, body mass index, depressive symptoms at 1^st^ follow-up, and general health status and sleep medication use at 4^th^ follow-up. Models with educational attainment as a moderator were additionally adjusted for household income, and models with household income as the moderator were additionally adjusted for educational attainment.

| Supplemental Table 2. Conditional indirect effect of major differential treatment on sleep dimensions through adaptive and maladaptive coping among non-Hispanic White women. | | | | | |
| --- | --- | --- | --- | --- | --- |
|  | | Conditional Indirect effect | | | |
| Mediator | Moderation effect on a path | *b* | SE | 95% Confidence Interval | |
| **Sleep duration** | | | | | |
| Different levels of Educational Attainment | | | | | |
| Adaptive Coping | ≤High school | -.00 | .00 | -.00 | .00 |
|  | Some college or technical degree | -.00 | .00 | -.00 | .00 |
|  | ≥college (bachelor’s or greater) | -.00 | .00 | -.00 | .00 |
| Index of moderated mediation: some college vs. ≤High school | | .00 | .00 | -.00 | .00 |
| Index of moderated mediation: ≥college vs. ≤High school | | .00 | .00 | -.00 | .00 |
| Maladaptive coping | ≤High school | -.00 | .00 | -.00 | .00 |
|  | Some college or technical degree | -.00 | .00 | -.00 | .00 |
|  | ≥college (bachelor’s or greater) | -.00 | .00 | -.00 | .00 |
| Index of moderated mediation: some college vs. ≤High school | | .00 | .00 | -.00 | .00 |
| Index of moderated mediation: ≥college vs. ≤High school | | .00 | .00 | -.00 | .00 |
|  | | | | | |
| Different levels of Household Income | | | | | |
| Adaptive Coping | <$50,000 | -.00 | .00 | -.00 | .00 |
|  | $50,000 - <$100,000 | -.00 | .00 | -.00 | .00 |
|  | ≥$100,000 | -.00 | .00 | -.00 | .00 |
| Index of moderated mediation: $50,000 - <$100,000 vs. <$50,000 | | .00 | .00 | -.00 | .00 |
| Index of moderated mediation: ≥$100,000 vs. <$50,000 | | -.00 | .00 | -.00 | .00 |
| Maladaptive coping | <$50,000 | -.00 | .00 | -.00 | .00 |
|  | $50,000 - <$100,000 | -.00 | .00 | -.00 | .00 |
|  | ≥$100,000 | -.00 | .00 | -.00 | .00 |
| Index of moderated mediation: $50,000 - <$100,000 vs. <$50,000 | | .00 | .00 | -.00 | .00 |
| Index of moderated mediation: ≥$100,000 vs. <$50,000 | | .00 | .00 | -.00 | .00 |
| **Poor Sleep Quality** | | | | | |
| Different levels of Educational Attainment | | | | | |
| Adaptive Coping | ≤High school | .01 | .01 | -.00 | .02 |
|  | Some college or technical degree | .00 | .00 | -.00 | .01 |
|  | ≥college (bachelor’s or greater) | .00 | .00 | -.00 | .01 |
| Index of moderated mediation: some college vs. ≤High school | | -.00 | .01 | -.02 | .01 |
| Index of moderated mediation: ≥college vs. ≤High school | | -.01 | .01 | -.02 | .01 |
| Maladaptive coping | ≤High school | .01 | .01 | -.01 | .04 |
|  | Some college or technical degree | .01 | .01 | .00 | .03 |
|  | ≥college (bachelor’s or greater) | .01 | .00 | .01 | .02 |
| Index of moderated mediation: some college vs. ≤High school | | .00 | .01 | -.02 | .02 |
| Index of moderated mediation: ≥college vs. ≤High school | | -.00 | .01 | -.02 | .02 |
|  | | | | | |
| Different levels of Household Income | | | | | |
| Adaptive Coping | <$50,000 | .00 | .00 | -.00 | .01 |
|  | $50,000 - <$100,000 | .00 | .00 | -.00 | .01 |
|  | ≥$100,000 | .00 | .00 | -.00 | .01 |
| Index of moderated mediation: $50,000 - <$100,000 vs. <$50,000 | | -.00 | .00 | -.01 | .01 |
| Index of moderated mediation: ≥$100,000 vs. <$50,000 | | .00 | .00 | -.01 | .01 |
| Maladaptive coping | <$50,000 | .02 | .01 | .01 | .03 |
|  | $50,000 - <$100,000 | .01 | .00 | -.00 | .01 |
|  | ≥$100,000 | .01 | .00 | .01 | .02 |
| Index of moderated mediation: $50,000 - <$100,000 vs. <$50,000 | | -.02 | .01 | -.03 | -.00 |
| Index of moderated mediation: ≥$100,000 vs. <$50,000 | | -.01 | .01 | -.02 | .00 |
| **Insomnia Symptoms** | | | | | |
| Different levels of Educational Attainment | | | | | |
| Adaptive Coping | ≤High school | .01 | .01 | -.01 | .03 |
|  | Some college or technical degree | .01 | .01 | -.00 | .02 |
|  | ≥college (bachelor’s or greater) | .00 | .00 | -.01 | .01 |
| Index of moderated mediation: some college vs. ≤High school | | -.01 | .01 | -.03 | .02 |
| Index of moderated mediation: ≥college vs. ≤High school | | -.01 | .01 | -.03 | .01 |
| Maladaptive coping | ≤High school | .03 | .02 | -.02 | .08 |
|  | Some college or technical degree | .03 | .01 | .00 | .05 |
|  | ≥college (bachelor’s or greater) | .02 | .01 | .01 | .04 |
| Index of moderated mediation: some college vs. ≤High school | | -.00 | .03 | -.05 | .05 |
| Index of moderated mediation: ≥college vs. ≤High school | | -.00 | .02 | -.05 | .04 |
|  | | | | | |
| Different levels of Household Income | | | | | |
| Adaptive Coping | <$50,000 | .00 | .01 | -.01 | .01 |
|  | $50,000 - <$100,000 | .00 | .00 | -.01 | .01 |
|  | ≥$100,000 | .00 | .00 | -.00 | .02 |
| Index of moderated mediation: $50,000 - <$100,000 vs. <$50,000 | | -.00 | .01 | -.02 | .01 |
| Index of moderated mediation: ≥$100,000 vs. <$50,000 | | -.00 | .01 | -.02 | .02 |
| Maladaptive coping | <$50,000 | .05 | .01 | .02 | .08 |
|  | $50,000 - <$100,000 | .01 | .01 | -.01 | .03 |
|  | ≥$100,000 | .03 | .01 | .01 | .05 |
| Index of moderated mediation: $50,000 - <$100,000 vs. <$50,000 | | -.04 | .02 | -.07 | -.01 |
| Index of moderated mediation: ≥$100,000 vs. <$50,000 | | -.02 | .02 | -.05 | .01 |

Note. All models adjusted for baseline age, marital status, employment status, body mass index, depressive symptoms at 1^st^ follow-up, and general health status and sleep medication use at 4^th^ follow-up. Models with educational attainment as a moderator were additionally adjusted for household income, and models with household income as the moderator were additionally adjusted for educational attainment.

| Supplemental Table 3. Conditional indirect effect of everyday differential treatment on sleep dimensions through adaptive and maladaptive coping among non-Hispanic Black women. | | | | | |
| --- | --- | --- | --- | --- | --- |
|  | | Conditional Indirect effect | | | |
| Mediator | Moderation effect on a path | *b* | SE | 95% Confidence Interval | |
| **Sleep duration** | | | | | |
| Different levels of Educational Attainment | | | | | |
| Adaptive Coping | ≤High school | .00 | .01 | -.01 | .01 |
|  | Some college or technical degree | -.00 | .00 | -.01 | .00 |
|  | ≥college (bachelor’s or greater) | .00 | .00 | -.00 | .00 |
| Index of moderated mediation: some college vs. ≤High school | | -.00 | .01 | -.02 | .01 |
| Index of moderated mediation: ≥college vs. ≤High school | | -.00 | .00 | -.01 | .01 |
| Maladaptive coping | ≤High school | .00 | .01 | -.01 | .02 |
|  | Some college or technical degree | .00 | .00 | -.00 | .01 |
|  | ≥college (bachelor’s or greater) | .00 | .00 | -.01 | .01 |
| Index of moderated mediation: some college vs. ≤High school | | -.00 | .01 | -.02 | .01 |
| Index of moderated mediation: ≥college vs. ≤High school | | -.00 | .01 | -.01 | .01 |
|  | | | | | |
| Different levels of Household Income | | | | | |
| Adaptive Coping | <$50,000 | -.00 | .00 | -.01 | .00 |
|  | $50,000 - <$100,000 | .00 | .00 | -.00 | .01 |
|  | ≥$100,000 | .00 | .00 | -.00 | .01 |
| Index of moderated mediation: $50,000 - <$100,000 vs. <$50,000 | | .00 | .00 | -.01 | .01 |
| Index of moderated mediation: ≥$100,000 vs. <$50,000 | | .00 | .00 | -.01 | .01 |
| Maladaptive coping | <$50,000 | .00 | .00 | -.01 | .01 |
|  | $50,000 - <$100,000 | .00 | .00 | -.00 | .01 |
|  | ≥$100,000 | .00 | .01 | -.01 | .02 |
| Index of moderated mediation: $50,000 - <$100,000 vs. <$50,000 | | -.00 | .00 | -.01 | .00 |
| Index of moderated mediation: ≥$100,000 vs. <$50,000 | | .00 | .00 | -.01 | .01 |
| **Poor Sleep Quality** | | | | | |
| Different levels of Educational Attainment | | | | | |
| Adaptive Coping | ≤High school | -.01 | .01 | -.03 | .01 |
|  | Some college or technical degree | .00 | .00 | -.01 | .01 |
|  | ≥college (bachelor’s or greater) | -.00 | .00 | -.01 | .00 |
| Index of moderated mediation: some college vs. ≤High school | | .01 | .01 | -.01 | .04 |
| Index of moderated mediation: ≥college vs. ≤High school | | .01 | .01 | -.01 | .03 |
| Maladaptive coping | ≤High school | .03 | .02 | -.00 | .06 |
|  | Some college or technical degree | .01 | .01 | -.01 | .03 |
|  | ≥college (bachelor’s or greater) | .01 | .01 | .00 | .03 |
| Index of moderated mediation: some college vs. ≤High school | | -.02 | .02 | -.06 | .01 |
| Index of moderated mediation: ≥college vs. ≤High school | | -.01 | .02 | -.05 | .03 |
|  | | | | | |
| Different levels of Household Income | | | | | |
| Adaptive Coping | <$50,000 | .00 | .01 | -.01 | .02 |
|  | $50,000 - <$100,000 | -.00 | .00 | -.01 | .00 |
|  | ≥$100,000 | -.00 | .01 | -.02 | .01 |
| Index of moderated mediation: $50,000 - <$100,000 vs. <$50,000 | | -.01 | .01 | -.03 | .00 |
| Index of moderated mediation: ≥$100,000 vs. <$50,000 | | -.01 | .01 | -.03 | .00 |
| Maladaptive coping | <$50,000 | .02 | .01 | -.00 | .04 |
|  | $50,000 - <$100,000 | .01 | .01 | -.01 | .02 |
|  | ≥$100,000 | .02 | .01 | .01 | .05 |
| Index of moderated mediation: $50,000 - <$100,000 vs. <$50,000 | | -.01 | .01 | -.04 | .01 |
| Index of moderated mediation: ≥$100,000 vs. <$50,000 | | .01 | .01 | -.02 | .04 |
| **Insomnia Symptoms** | | | | | |
| Different levels of Educational Attainment | | | | | |
| Adaptive Coping | ≤High school | -.00 | .01 | -.04 | .02 |
|  | Some college or technical degree | .00 | .01 | -.01 | .01 |
|  | ≥college (bachelor’s or greater) | -.00 | .01 | -.02 | .01 |
| Index of moderated mediation: some college vs. ≤High school | | .00 | .02 | -.03 | .05 |
| Index of moderated mediation: ≥college vs. ≤High school | | .00 | .01 | -.02 | .03 |
| Maladaptive coping | ≤High school | .07 | .05 | -.01 | .17 |
|  | Some college or technical degree | .01 | .03 | -.04 | .07 |
|  | ≥college (bachelor’s or greater) | .04 | .02 | .01 | .08 |
| Index of moderated mediation: some college vs. ≤High school | | -.05 | .05 | -.17 | .04 |
| Index of moderated mediation: ≥college vs. ≤High school | | -.03 | .04 | -.12 | .06 |
|  | | | | | |
| Different levels of Household Income | | | | | |
| Adaptive Coping | <$50,000 | .00 | .01 | -.01 | .03 |
|  | $50,000 - <$100,000 | -.00 | .01 | -.02 | .01 |
|  | ≥$100,000 | -.00 | .01 | -.03 | .01 |
| Index of moderated mediation: $50,000 - <$100,000 vs. <$50,000 | | -.00 | .01 | -.04 | .02 |
| Index of moderated mediation: ≥$100,000 vs. <$50,000 | | -.00 | .02 | -.04 | .02 |
| Maladaptive coping | <$50,000 | .04 | .03 | -.01 | .10 |
|  | $50,000 - <$100,000 | .02 | .02 | -.02 | .05 |
|  | ≥$100,000 | .07 | .03 | .02 | .14 |
| Index of moderated mediation: $50,000 - <$100,000 vs. <$50,000 | | -.02 | .03 | -.09 | .04 |
| Index of moderated mediation: ≥$100,000 vs. <$50,000 | | .03 | .04 | -.03 | .11 |

Note. All models adjusted for baseline age, marital status, employment status, body mass index, depressive symptoms at 1^st^ follow-up, and general health status and sleep medication use at 4^th^ follow-up. Models with educational attainment as a moderator were additionally adjusted for household income, and models with household income as the moderator were additionally adjusted for educational attainment.

| Supplemental Table 4. Conditional indirect effect of major differential treatment on sleep dimensions through adaptive and maladaptive coping among non-Hispanic Black women. | | | | | |
| --- | --- | --- | --- | --- | --- |
|  | | Conditional Indirect effect | | | |
| Mediator | Moderation effect on a path | *b* | SE | 95% Confidence Interval | |
| **Sleep duration** | | | | | |
| Different levels of Educational Attainment | | | | | |
| Adaptive Coping | ≤High school | -.00 | .00 | -.01 | .01 |
|  | Some college or technical degree | .00 | .00 | -.00 | .00 |
|  | ≥college (bachelor’s or greater) | -.00 | .00 | -.00 | .00 |
| Index of moderated mediation: some college vs. ≤High school | | .00 | .00 | -.01 | .01 |
| Index of moderated mediation: ≥college vs. ≤High school | | .00 | .00 | -.01 | .01 |
| Maladaptive coping | ≤High school | .00 | .01 | -.01 | .02 |
|  | Some college or technical degree | .00 | .00 | -.00 | .01 |
|  | ≥college (bachelor’s or greater) | .00 | .00 | -.00 | .00 |
| Index of moderated mediation: some college vs. ≤High school | | -.00 | .01 | -.02 | .01 |
| Index of moderated mediation: ≥college vs. ≤High school | | -.00 | .01 | -.02 | .01 |
|  | | | | | |
| Different levels of Household Income | | | | | |
| Adaptive Coping | <$50,000 | .00 | .00 | -.00 | .01 |
|  | $50,000 - <$100,000 | -.00 | .00 | -.01 | .00 |
|  | ≥$100,000 | .00 | .00 | -.00 | .00 |
| Index of moderated mediation: $50,000 - <$100,000 vs. <$50,000 | | -.00 | .00 | -.01 | .01 |
| Index of moderated mediation: ≥$100,000 vs. <$50,000 | | .00 | .00 | -.01 | .00 |
| Maladaptive coping | <$50,000 | .00 | .00 | -.00 | .01 |
|  | $50,000 - <$100,000 | .00 | .00 | -.00 | .01 |
|  | ≥$100,000 | .00 | .00 | -.00 | .01 |
| Index of moderated mediation: $50,000 - <$100,000 vs. <$50,000 | | .00 | .00 | -.01 | .01 |
| Index of moderated mediation: ≥$100,000 vs. <$50,000 | | .00 | .00 | -.01 | .01 |
| **Poor Sleep Quality** | | | | | |
| Different levels of Educational Attainment | | | | | |
| Adaptive Coping | ≤High school | .00 | .01 | -.02 | .02 |
|  | Some college or technical degree | -.00 | .00 | -.01 | .01 |
|  | ≥college (bachelor’s or greater) | .00 | .00 | -.00 | .01 |
| Index of moderated mediation: some college vs. ≤High school | | -.00 | .01 | -.02 | .02 |
| Index of moderated mediation: ≥college vs. ≤High school | | -.00 | .01 | -.02 | .02 |
| Maladaptive coping | ≤High school | .02 | .02 | -.01 | .06 |
|  | Some college or technical degree | .00 | .01 | -.01 | .02 |
|  | ≥college (bachelor’s or greater) | .00 | .00 | -.01 | .01 |
| Index of moderated mediation: some college vs. ≤High school | | -.02 | .02 | -.06 | .01 |
| Index of moderated mediation: ≥college vs. ≤High school | | -.02 | .02 | -.06 | .01 |
|  | | | | | |
| Different levels of Household Income | | | | | |
| Adaptive Coping | <$50,000 | -.00 | .00 | -.01 | .01 |
|  | $50,000 - <$100,000 | .00 | .00 | -.00 | .01 |
|  | ≥$100,000 | -.00 | .00 | -.01 | .00 |
| Index of moderated mediation: $50,000 - <$100,000 vs. <$50,000 | | .01 | .01 | -.00 | .02 |
| Index of moderated mediation: ≥$100,000 vs. <$50,000 | | .00 | .01 | -.01 | .01 |
| Maladaptive coping | <$50,000 | -.00 | .01 | -.02 | .01 |
|  | $50,000 - <$100,000 | .01 | .01 | -.00 | .02 |
|  | ≥$100,000 | .00 | .01 | -.01 | .02 |
| Index of moderated mediation: $50,000 - <$100,000 vs. <$50,000 | | .01 | .01 | -.01 | .03 |
| Index of moderated mediation: ≥$100,000 vs. <$50,000 | | .01 | .01 | -.01 | .03 |
| **Insomnia Symptoms** | | | | | |
| Different levels of Educational Attainment | | | | | |
| Adaptive Coping | ≤High school | .00 | .01 | -.02 | .02 |
|  | Some college or technical degree | .00 | .01 | -.01 | .01 |
|  | ≥college (bachelor’s or greater) | .00 | .00 | -.01 | .01 |
| Index of moderated mediation: some college vs. ≤High school | | -.00 | .01 | -.03 | .02 |
| Index of moderated mediation: ≥college vs. ≤High school | | -.00 | .01 | -.02 | .02 |
| Maladaptive coping | ≤High school | .06 | .04 | -.02 | .15 |
|  | Some college or technical degree | .02 | .02 | -.02 | .06 |
|  | ≥college (bachelor’s or greater) | .01 | .01 | -.02 | .03 |
| Index of moderated mediation: some college vs. ≤High school | | -.04 | .05 | -.14 | .05 |
| Index of moderated mediation: ≥college vs. ≤High school | | -.05 | .05 | -.15 | .03 |
|  | | | | | |
| Different levels of Household Income | | | | | |
| Adaptive Coping | <$50,000 | -.00 | .01 | -.02 | .01 |
|  | $50,000 - <$100,000 | .00 | .01 | -.01 | .02 |
|  | ≥$100,000 | -.00 | .01 | -.02 | .01 |
| Index of moderated mediation: $50,000 - <$100,000 vs. <$50,000 | | .00 | .01 | -.02 | .03 |
| Index of moderated mediation: ≥$100,000 vs. <$50,000 | | .00 | .01 | -.02 | .02 |
| Maladaptive coping | <$50,000 | -.00 | .02 | -.05 | .05 |
|  | $50,000 - <$100,000 | .02 | .01 | -.01 | .05 |
|  | ≥$100,000 | .02 | .02 | -.02 | .05 |
| Index of moderated mediation: $50,000 - <$100,000 vs. <$50,000 | | .02 | .03 | --.04 | .08 |
| Index of moderated mediation: ≥$100,000 vs. <$50,000 | | .02 | .03 | -.04 | .08 |

Note. All models adjusted for baseline age, marital status, employment status, body mass index, depressive symptoms at 1^st^ follow-up, and general health status and sleep medication use at 4^th^ follow-up. Models with educational attainment as a moderator were additionally adjusted for household income, and models with household income as the moderator were additionally adjusted for educational attainment.

| Supplemental Table 5. Conditional indirect effect of everyday differential treatment on sleep dimensions through adaptive and maladaptive coping among Hispanic/Latina women. | | | | | |
| --- | --- | --- | --- | --- | --- |
|  | | Conditional Indirect effect | | | |
| Mediator | Moderation effect on a path | *b* | SE | 95% Confidence Interval | |
| **Sleep duration** | | | | | |
| Different levels of Educational Attainment | | | | | |
| Adaptive Coping | ≤High school | -.01 | .01 | -.03 | .00 |
|  | Some college or technical degree | .00 | .01 | -.01 | .02 |
|  | ≥college (bachelor’s or greater) | -.00 | .00 | -.01 | .00 |
| Index of moderated mediation: some college vs. ≤High school | | .01 | .01 | -.01 | .04 |
| Index of moderated mediation: ≥college vs. ≤High school | | .01 | .01 | -.01 | .03 |
| Maladaptive coping | ≤High school | -.00 | .01 | -.02 | .01 |
|  | Some college or technical degree | -.00 | .01 | -.02 | .00 |
|  | ≥college (bachelor’s or greater) | .00 | .00 | -.00 | .01 |
| Index of moderated mediation: some college vs. ≤High school | | -.00 | .01 | -.02 | .01 |
| Index of moderated mediation: ≥college vs. ≤High school | | .01 | .01 | -.01 | .03 |
|  | | | | | |
| Different levels of Household Income | | | | | |
| Adaptive Coping | <$50,000 | -.00 | .01 | -.02 | .00 |
|  | $50,000 - <$100,000 | .00 | .00 | -.01 | .01 |
|  | ≥$100,000 | -.01 | .01 | -.02 | .00 |
| Index of moderated mediation: $50,000 - <$100,000 vs. <$50,000 | | .01 | .01 | -.01 | .03 |
| Index of moderated mediation: ≥$100,000 vs. <$50,000 | | -.00 | .01 | -.02 | .01 |
| Maladaptive coping | <$50,000 | -.00 | .00 | -.01 | .01 |
|  | $50,000 - <$100,000 | -.00 | .00 | -.01 | .00 |
|  | ≥$100,000 | .00 | .01 | -.00 | .02 |
| Index of moderated mediation: $50,000 - <$100,000 vs. <$50,000 | | -.00 | .01 | -.01 | .01 |
| Index of moderated mediation: ≥$100,000 vs. <$50,000 | | .01 | .01 | -.00 | .03 |
| **Poor Sleep Quality** | | | | | |
| Different levels of Educational Attainment | | | | | |
| Adaptive Coping | ≤High school | -.00 | .01 | -.02 | .02 |
|  | Some college or technical degree | .00 | .00 | -.01 | .01 |
|  | ≥college (bachelor’s or greater) | -.00 | .00 | -.01 | .01 |
| Index of moderated mediation: some college vs. ≤High school | | .00 | .01 | -.02 | .02 |
| Index of moderated mediation: ≥college vs. ≤High school | | .00 | .01 | -.02 | .02 |
| Maladaptive coping | ≤High school | -.01 | .01 | -.03 | .01 |
|  | Some college or technical degree | -.01 | .01 | -.03 | .00 |
|  | ≥college (bachelor’s or greater) | .00 | .01 | -.01 | .02 |
| Index of moderated mediation: some college vs. ≤High school | | -.00 | .01 | -.03 | .02 |
| Index of moderated mediation: ≥college vs. ≤High school | | .01 | .01 | -.01 | .04 |
|  | | | | | |
| Different levels of Household Income | | | | | |
| Adaptive Coping | <$50,000 | -.00 | .00 | -.01 | .01 |
|  | $50,000 - <$100,000 | .00 | .00 | -.01 | .01 |
|  | ≥$100,000 | -.00 | .01 | -.01 | .01 |
| Index of moderated mediation: $50,000 - <$100,000 vs. <$50,000 | | .00 | .01 | -.01 | .01 |
| Index of moderated mediation: ≥$100,000 vs. <$50,000 | | -.00 | .00 | -.01 | .01 |
| Maladaptive coping | <$50,000 | -.00 | .01 | -.02 | .01 |
|  | $50,000 - <$100,000 | -.00 | .01 | -.02 | .00 |
|  | ≥$100,000 | .01 | .01 | -.01 | .02 |
| Index of moderated mediation: $50,000 - <$100,000 vs. <$50,000 | | -.00 | .01 | -.02 | .01 |
| Index of moderated mediation: ≥$100,000 vs. <$50,000 | | .01 | .01 | -.01 | .03 |
| **Insomnia Symptoms** | | | | | |
| Different levels of Educational Attainment | | | | | |
| Adaptive Coping | ≤High school | .01 | .03 | -.06 | .08 |
|  | Some college or technical degree | -.00 | .01 | -.03 | .03 |
|  | ≥college (bachelor’s or greater) | .00 | .01 | -.02 | .03 |
| Index of moderated mediation: some college vs. ≤High school | | -.01 | .04 | -.10 | .08 |
| Index of moderated mediation: ≥college vs. ≤High school | | -.00 | .03 | -.07 | .05 |
| Maladaptive coping | ≤High school | -.04 | .05 | -.15 | .05 |
|  | Some college or technical degree | -.05 | .03 | -.12 | .01 |
|  | ≥college (bachelor’s or greater) | .02 | .02 | -.03 | .07 |
| Index of moderated mediation: some college vs. ≤High school | | -.00 | .06 | -.12 | .12 |
| Index of moderated mediation: ≥college vs. ≤High school | | .06 | .06 | -.04 | .19 |
|  | | | | | |
| Different levels of Household Income | | | | | |
| Adaptive Coping | <$50,000 | .00 | .02 | -.03 | .04 |
|  | $50,000 - <$100,000 | -.00 | .01 | -.03 | .03 |
|  | ≥$100,000 | .00 | .02 | -.05 | .05 |
| Index of moderated mediation: $50,000 - <$100,000 vs. <$50,000 | | -.00 | .02 | -.05 | .05 |
| Index of moderated mediation: ≥$100,000 vs. <$50,000 | | .00 | .02 | -.04 | .04 |
| Maladaptive coping | <$50,000 | -.01 | .03 | -.08 | .04 |
|  | $50,000 - <$100,000 | -.02 | .03 | -.08 | .02 |
|  | ≥$100,000 | .03 | .03 | -.02 | .09 |
| Index of moderated mediation: $50,000 - <$100,000 vs. <$50,000 | | -.01 | .03 | -.08 | .07 |
| Index of moderated mediation: ≥$100,000 vs. <$50,000 | | .04 | .04 | -.03 | .13 |

Note. All models adjusted for baseline age, marital status, employment status, body mass index, depressive symptoms at 1^st^ follow-up, and general health status and sleep medication use at 4^th^ follow-up. Models with educational attainment as a moderator were additionally adjusted for household income, and models with household income as the moderator were additionally adjusted for educational attainment.

| Supplemental Table 6. Conditional indirect effect of major differential treatment on sleep dimensions through adaptive and maladaptive coping among Hispanic/Latina women. | | | | | |
| --- | --- | --- | --- | --- | --- |
|  | | Conditional Indirect effect | | | |
| Mediator | Moderation effect on a path | *b* | SE | 95% Confidence Interval | |
| **Sleep duration** | | | | | |
| Different levels of Educational Attainment | | | | | |
| Adaptive Coping | ≤High school | -.02 | .02 | -.06 | .00 |
|  | Some college or technical degree | .01 | .01 | -.00 | .03 |
|  | ≥college (bachelor’s or greater) | .01 | .01 | -.00 | .02 |
| Index of moderated mediation: some college vs. ≤High school | | .02 | .02 | -.01 | .08 |
| Index of moderated mediation: ≥college vs. ≤High school | | .03 | .02 | -.00 | .07 |
| Maladaptive coping | ≤High school | -.00 | .01 | -.02 | .02 |
|  | Some college or technical degree | -.01 | .01 | -.02 | .01 |
|  | ≥college (bachelor’s or greater) | -.00 | .00 | -.01 | .01 |
| Index of moderated mediation: some college vs. ≤High school | | -.01 | .01 | -.04 | .01 |
| Index of moderated mediation: ≥college vs. ≤High school | | -.00 | .01 | -.03 | .02 |
|  | | | | | |
| Different levels of Household Income | | | | | |
| Adaptive Coping | <$50,000 | -.00 | .01 | -.02 | .01 |
|  | $50,000 - <$100,000 | .01 | .01 | -.00 | .03 |
|  | ≥$100,000 | .00 | .01 | -.01 | .02 |
| Index of moderated mediation: $50,000 - <$100,000 vs. <$50,000 | | .01 | .01 | -.00 | .04 |
| Index of moderated mediation: ≥$100,000 vs. <$50,000 | | .00 | .01 | -.01 | .02 |
| Maladaptive coping | <$50,000 | .00 | .01 | -.01 | .02 |
|  | $50,000 - <$100,000 | -.01 | .01 | -.02 | .00 |
|  | ≥$100,000 | .00 | .01 | -.01 | .02 |
| Index of moderated mediation: $50,000 - <$100,000 vs. <$50,000 | | -.01 | .01 | -.03 | .01 |
| Index of moderated mediation: ≥$100,000 vs. <$50,000 | | .00 | .01 | -.01 | .03 |
| **Poor Sleep Quality** | | | | | |
| Different levels of Educational Attainment | | | | | |
| Adaptive Coping | ≤High school | -.00 | .01 | -.03 | .02 |
|  | Some college or technical degree | .00 | .01 | -.02 | .02 |
|  | ≥college (bachelor’s or greater) | .00 | .01 | -.01 | .01 |
| Index of moderated mediation: some college vs. ≤High school | | .00 | .02 | -.04 | .04 |
| Index of moderated mediation: ≥college vs. ≤High school | | .00 | .02 | -.03 | .03 |
| Maladaptive coping | ≤High school | .01 | .02 | -.02 | .05 |
|  | Some college or technical degree | -.01 | .01 | -.04 | .01 |
|  | ≥college (bachelor’s or greater) | -.00 | .01 | -.02 | .01 |
| Index of moderated mediation: some college vs. ≤High school | | -.02 | .02 | -.07 | .02 |
| Index of moderated mediation: ≥college vs. ≤High school | | -.01 | .02 | -.05 | .03 |
|  | | | | | |
| Different levels of Household Income | | | | | |
| Adaptive Coping | <$50,000 | -.00 | .00 | -.01 | .01 |
|  | $50,000 - <$100,000 | .00 | .01 | -.01 | .01 |
|  | ≥$100,000 | .00 | .00 | -.01 | .01 |
| Index of moderated mediation: $50,000 - <$100,000 vs. <$50,000 | | .00 | .01 | -.02 | .02 |
| Index of moderated mediation: ≥$100,000 vs. <$50,000 | | .00 | .01 | -.01 | .01 |
| Maladaptive coping | <$50,000 | .00 | .01 | -.01 | .03 |
|  | $50,000 - <$100,000 | -.01 | .01 | -.03 | .00 |
|  | ≥$100,000 | .01 | .01 | -.01 | .03 |
| Index of moderated mediation: $50,000 - <$100,000 vs. <$50,000 | | -.02 | .02 | -.06 | .01 |
| Index of moderated mediation: ≥$100,000 vs. <$50,000 | | .00 | .01 | -.03 | .03 |
| **Insomnia Symptoms** | | | | | |
| Different levels of Educational Attainment | | | | | |
| Adaptive Coping | ≤High school | .01 | .04 | -.08 | .10 |
|  | Some college or technical degree | -.00 | .02 | -.06 | .05 |
|  | ≥college (bachelor’s or greater) | -.00 | .02 | -.04 | .03 |
| Index of moderated mediation: some college vs. ≤High school | | -.01 | .07 | -.15 | .12 |
| Index of moderated mediation: ≥college vs. ≤High school | | -.01 | .06 | -.13 | .10 |
| Maladaptive coping | ≤High school | .00 | .08 | -.11 | .20 |
|  | Some college or technical degree | -.06 | .05 | -.16 | .03 |
|  | ≥college (bachelor’s or greater) | -.00 | .03 | -.07 | .06 |
| Index of moderated mediation: some college vs. ≤High school | | -.09 | .09 | -.29 | .07 |
| Index of moderated mediation: ≥college vs. ≤High school | | -.04 | .08 | -.21 | .12 |
|  | | | | | |
| Different levels of Household Income | | | | | |
| Adaptive Coping | <$50,000 | .00 | .01 | -.02 | .03 |
|  | $50,000 - <$100,000 | -.00 | .02 | -.05 | .05 |
|  | ≥$100,000 | .00 | .01 | -.03 | .03 |
| Index of moderated mediation: $50,000 - <$100,000 vs. <$50,000 | | -.00 | .03 | -.07 | .06 |
| Index of moderated mediation: ≥$100,000 vs. <$50,000 | | -.00 | .02 | -.04 | .04 |
| Maladaptive coping | <$50,000 | .02 | .05 | -.07 | .14 |
|  | $50,000 - <$100,000 | -.05 | .04 | -.14 | .00 |
|  | ≥$100,000 | .03 | .04 | -.04 | .12 |
| Index of moderated mediation: $50,000 - <$100,000 vs. <$50,000 | | -.08 | .07 | -.23 | .03 |
| Index of moderated mediation: ≥$100,000 vs. <$50,000 | | .01 | .06 | -.13 | .13 |

Note. All models adjusted for baseline age, marital status, employment status, body mass index, depressive symptoms at 1^st^ follow-up, and general health status and sleep medication use at 4^th^ follow-up. Models with educational attainment as a moderator were additionally adjusted for household income, and models with household income as the moderator were additionally adjusted for educational attainment.
